# Evaluating Pediatric Reference Ranges for Extended Immunophenotyping from a Finnish Cohort against Published References

**DOI:** 10.1007/s10875-025-01959-y

**Published:** 2025-11-18

**Authors:** Elli Äärimaa, Anssi Kesäläinen, Samuel Askeli, Anne Toivonen, Okko Savonius, Oscar Brück, Pauliina Lusila, Kim Vettenranta, Santtu Heinonen, Timo Jahnukainen, Minna Koskenvuo, Sanna Siitonen, Sari Lehtimäki, Eliisa Kekäläinen

**Affiliations:** 1 Translational Immunology Research Program, University of Helsinki, Helsinki, 00014 Finland; 2 Clinical Microbiology, HUS Diagnostic Center, Helsinki University Hospital and University of Helsinki, Helsinki, Finland; 3 Department of Pediatric Nephrology and Transplantation, New Children’s Hospital, Pediatric Research Center, Helsinki University Hospital and University of Helsinki, Helsinki, Finland; 4 Hematoscope Lab, Comprehensive Cancer Center, Department of Clinical Chemistry, HUS Diagnostic Center, Helsinki University Hospital and University of Helsinki, Helsinki, Finland; 5 New Children’s Hospital, Pediatric Research Center, Helsinki University Hospital and University of Helsinki, Helsinki, Finland; 6 FVR, Finnish Vaccine Research, Tampere, Finland; 7 Division of Pediatric Hematology, Oncology and Stem Cell Transplantation, New Children’s Hospital, Helsinki University Hospital and University of Helsinki, Helsinki, Finland; 8 Clinical Chemistry, HUS Diagnostic Center, Helsinki University Hospital and University of Helsinki, Helsinki, Finland

**Keywords:** Reference values, Lymphocyte subsets, Pediatrics, Flow cytometry, Immunophenotyping, Immunodeficiency

## Abstract

Flow cytometric immunophenotyping of lymphocytes and dendritic cells, and functional lymphocyte mitogen response tests are used in the diagnostics of inborn errors of immunity (IEI), especially in pediatrics. These routinely used tests lack sufficient age-matched reference values in children. We established reference values for lymphocyte and dendritic cell subsets for four age groups from 68 healthy children under 12 years of age. These values were then compared to prior publicly available articles and 46 clinical samples from children with confirmed IEI diagnosis. Mitogen response results were also compared between 27 children and 177 adults. In the literature review, we found considerable variability in lymphocyte subset definitions and statistical approaches. Most IEI patients had increased transitional and naïve B, and decreased memory B cells. CHH patients had increased γδ T and DNTs. Lymphocyte stimulation via FASCIA method provides weaker stimulation results in children than in adults, which seems to result from a larger proportional count of naïve lymphocytes in children. The established reference values can be used in diagnostics of pediatric immunological conditions in laboratories that use similar analytic methods. Lower lymphocyte mitogen response results in children need to be taken into consideration when interpreting the results of lymphocyte functional tests.

**Supplementary Information:**

The online version contains supplementary material available at 10.1007/s10875-025-01959-y.

## Introduction

Determining the compositions of T and B lymphocyte compartments via flow cytometric analysis remains the quintessential method in the diagnostics of immune-mediated conditions such as inborn errors of immunity (IEI) and acquired immunodeficiencies [1–3]. Extended immunophenotyping of lymphocyte subsets provides crucial information about the counts, relations, and activation of these cells. The composition of T and B cell compartments changes drastically during childhood [4–6]. The levels of naïve T cells and recent thymic emigrants (RTEs) decrease significantly after early childhood as the thymopoiesis decelerates whereas the counts of memory cells increase as the immune system matures and encounters pathogens [7, 8]. As IEIs are more prevalent in children and the lymphocyte compartment evolves constantly during childhood, age-matched pediatric reference values for lymphocyte subsets are necessary for more precise diagnostics.

Reference values for peripheral blood lymphocyte subpopulations have been published in previous studies, however only a few studies have provided values for more recently discovered subpopulations frequently used in clinical diagnostics. Specifically, RTE cells, which are currently used for assessing the level of thymopoiesis, and T cell receptor alpha-beta positive double-negative T cells (TCRαβ^+^CD4^−^CD8^−^, DNT cells), which show increased counts in autoimmune processes (e.g. autoimmune lymphoproliferative syndrome, ALPS [9]), lack proper age-matched reference values for children.

The aim of this study was to determine reference values for lymphocyte subpopulations and dendritic cells for healthy children aged 0–12 years. Our reference values include all T and B cell subpopulations currently used in the diagnostics of immunodeficiencies in children. We also compared our reference values with results from children diagnosed with an immunodeficiency syndrome and conducted a review on the literature of pediatric reference values for lymphocyte immunophenotyping to provide further validation to our reference values and to estimate how well the assessed reference values would fare in clinical practice. Additionally, we derived suggestive reference values for under-9-year-old children for flow-cytometric assay for specific cell-mediated immune-response in activated whole blood (FASCIA) and performed correlation analyses with FASCIA results and T lymphocyte subpopulations.

## Methods

### Study Populations and Sample Collection

Peripheral venous blood samples were collected from a convenience cohort of 68 Finnish children aged 0–12 years (3 months to 11 years and 7 months) without suspected immunological abnormalities and who were undergoing elective procedures or imaging in the New Children’s Hospital, Helsinki, Finland between May 2018 and May 2024. Of the children, 41 (60%) were male and 27 (40%) female. Children were divided into four age cohorts (0–2, 2–4, 4–6, 6–12 years) to determine age-matched reference intervals and medians. Age cohort of 0–2 years was chosen as our sample size of less than 1-year-olds was limited (*n* = 6). To ensure that the participants were immunologically healthy, a manual review of the medical records was performed and the guardians interviewed for participants’ history of cancer, autoimmune diseases, recurrent infections, or immunosuppressive medications. After recruitment and sample collection, both samples and data were stored and analyzed as anonymized. Data about the ethnicity of the participants was not collected, but majority of the cohort were of Finnish ethnicity.

Lymphocyte stimulation response test via FASCIA method was performed for a subgroup of 27 healthy children. FASCIA samples were collected as a convenience series of control individuals sampled on specific dates when lymphocyte stimulation tests were performed as part of the laboratory routine. Median age was 3.0 years (0.7 − 8.9 years) and 16 were male (59%) and 11 were female (41%). Pediatric FASCIA values were compared to samples from 177 adults that were blood donors for Finnish Red Cross Blood Service. Age and sex were reported for 96 adults with 64 males (67%) and 32 females (33%), and the median age being 55 years (19 − 70 years). All blood samples for lymphocyte immunophenotyping and stimulation via FASCIA were analyzed with flow cytometry in the HUS Diagnostic Center, Clinical Microbiology & Clinical Chemistry, Helsinki, Finland.

We acquired immunophenotyping data of IEI patients from the HUS patient registry. Data were available for 252 patients aged 1 − 12 years and who resided in the Hospital District of Helsinki and Uusimaa (HUS). Samples from children under the age of 1 were limited and not included in the analysis. A total of 49 patients were selected based on a definitive IEI diagnosis, as indicated by a physician-assigned IEI diagnosis with a matching ICD-10 code. Patients were divided into five cohorts based on their main IEI diagnosis: 22q11.2 deletion syndrome (22q11.2del, ICD-10: D82.1), cartilage-hair hypoplasia (CHH, Q77.82), combined immunodeficiency (CID, D81), common variable immunodeficiency (CVID, D83) or antibody deficiency (D80), and hyperimmunoglobulin E syndrome (HIES, D82.4). Three patients with immunosuppressive medications (mycophenolic acid and rituximab) were excluded, resulting in 46 patients. IEI patients were then compared to the age-matched reference values of key lymphocyte subsets which consisted of proportional values of naïve CD4^+^ and CD8^+^, RTE, CD8^+^ TEMRA, DNT, γδ T cells and all B cell subsets. T cell data was available for all IEI cohorts and B cell data only for CID, CVID or antibody deficiency, and HIES cohorts. The patient selection algorithm is included in Supplementary Figure S1. 50% of IEI patients were female (*n* = 23). None of the included patients had undergone hematopoietic stem cell transplantation (HSCT) nor had a cancer diagnosis (ICD-10: C00 − D49).

### Flow Cytometry, Cell Subset Definitions and FASCIA Method

Flow cytometric analyses of blood samples were conducted using FACS Canto or FACS Lyric systems (BD Biosciences). Detailed sample processing protocol is shown in Supplementary Materials as well as cell surface markers used to define lymphocyte and dendritic cell subpopulations (Supplementary Table S1), monoclonal antibodies, fluorochromes, clones (Supplementary Table S2) and gating strategies (Supplementary Figures S2–S4) used for the flow cytometric analysis. For a subgroup of 25 children, absolute lymphocyte values provided by calculation from Sysmex hematological analyzer were compared with values from flow cytometric BD FACS Lyric system with BD Trucount tubes (Supplementary Figure S5). The absolute values were determined for cell subsets where IEI diagnostic criteria of the ESID Registry [10] specifically mention absolute counts (naïve T cells and RTEs).

Flow-cytometric assay for specific cell-mediated immune-response in activated whole blood (FASCIA) assesses the mitogenic activity of lymphocytes by cell size detection instead of radioactive markers. The overall stimulation responses of lymphocytes were assessed by the mean of percentages of activated cells per mitogen (phytohemagglutinin, PHA; Concavalin A, ConA), resulting in FASCIA score (value of 0 − 100). Detailed sample processing protocol is shown in Supplementary Materials.

### Statistical Analysis

Outliers from the lymphocyte subset and stimulation data were identified using Dixon’s criteria and the normality of the distributions for each age-matched cell subpopulation was investigated with Shapiro–Wilk’s test. As all data were not normally distributed, reference intervals were determined non-parametrically as 10th and 90th percentiles to avoid normality assumptions. For the adult FASCIA population, the 5th percentile (with 90% CI) was used for the lower reference limit. Kruskal–Wallis test was used to test the differences of reference intervals and medians between the age groups. Wilcoxon rank sum test with multiple comparisons correction with Benjamini–Hochberg (FDR) test was used to compare the results between sexes. Spearman’s correlation coefficient was used to assess the correlation between FASCIA-score and pediatric lymphocyte subsets. Statistical analyses were performed using R software (version 4.4.1) [11], MS Excel (Microsoft Excel 16.78) and Graph Pad Prism (version 9.5.1). P-values < 0.05 were considered statistically significant.

For the continuous reference range model of RTEs, a quantile regression was applied to log-transformed RTE data that was modelled at multiple percentiles (2.5% – 97.5%) and predictions were generated across a continuous age range. Results were then transformed back to the original scale.

## Results

### Assessment of Age-matched Reference Values

**Fig. 1:**
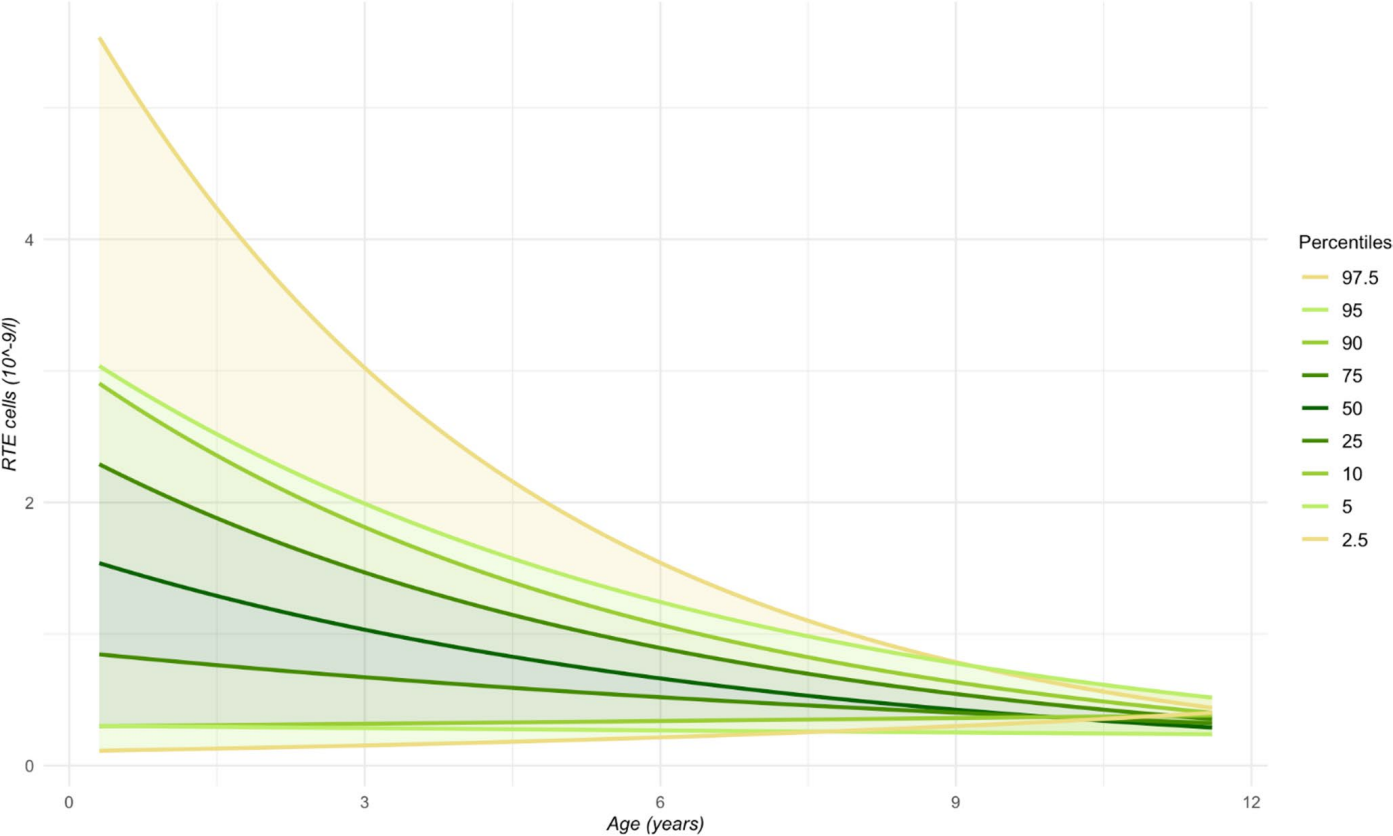
Continuous reference interval model for recent thymic emigrant (RTE) cell count. The estimation is based on 68 samples from children aged 0 − 12 years. As only 6 participants were under 1 year of age, the estimations among the newborn and infants are limited

Table 1 shows the reference values for pediatric T lymphocyte subsets. Total lymphocyte count decreased with age, as well as most T lymphocyte subpopulations. The counts of naive T cells decreased whereas memory T cells [total, effector memory (TEM) and central memory (TCM), as well as terminally differentiated effector (TEMRA)] increased. The B lymphocyte compartment (Table 2) followed the same pattern, with naive cells being more pronounced in early childhood, while the count of memory cells increased later in life. As expected, transitional B lymphocytes also decreased during childhood while there was no significant change in the level of plasmablasts. The dendritic cell compartment remained relatively constant during childhood. After multiple comparisons corrections, the differences between sexes were not statistically significant, albeit our sample size limits our ability to estimate sex-related differences reliably. Continuous reference range model for the absolute count of RTE cells is shown in Fig. 1. Our model shows an exponential decay of RTE cells during childhood. As there was a limited number of participants under one year of age, the reliability of this continuous model in newborns and infants is restricted. The absolute counts of the main T cell populations underwent dual-platform analysis (Sysmex calculation and flow-cytometric Trucount analysis) where we found that the flow-cytometric method provides slightly higher values than calculation from Sysmex analyzer (Supplementary Figure S5).

**Table 1:** Reference values for T lymphocyte subsets for children aged 0 − 12 years. Percentages (%), and absolute cell counts (x10^9^/l) when available, are shown. Counts are shown as median and percentile range (P10 − P90)

	0–2 years (median: 1.1) *n* = 23	2–4 years (median: 3.4) *n* = 14	4–6 years (median: 4.9) *n* = 16	6–12 years (median: 8.1) *n* = 15
Total lymphocyte count (x10^9^/l)	5.32 (2.91 − 7.33)	2.73 (1.93 − 5.36)	2.57 (1.89 − 4.54)	1.97 (1.9 − 2.73)
CD3^+^ lymphocytes (x10^9^/l)	3.86 (1.67 − 4.83)	1.81 (1.31 − 3.59)	1.98 (1.29 − 3.23)	1.45 (1.25 − 2.12)
CD3^+^ lymphocytes (%)^a^	67 (56 − 78)	65 (62 − 71)	74 (64 − 80)	71 (63 − 75)
Naive T lymphocytes (x10^9^/l)	3.05 (1.3 − 4)	1.4 (0.96 − 2.64)	1.48 (0.75 − 2.35)	0.92 (0.73 − 1.36)
Naive T lymphocytes (%)^b^	81 (71 − 87)	72 (68 − 81)	72 (57 − 80)	61 (56 − 71)
Memory T lymphocytes (%)^b^	10 (6 − 15)	17 (11 − 20)	18 (13 − 27)	22 (16 − 28)
HLA-DR^+^ CD38^+^ activated T lymphocytes (%)^b^	4 (3 − 7)	5 (3 − 10)	5 (3 − 6)	4 (3 − 9)
TCRαβ lymphocytes (%)^b^	96 (93 − 97)	93 (90 − 96)	92 (86 − 95)	91 (83 − 95)
TCRγδ lymphocytes (%)^b^	4 (3 − 7)	7 (4 − 10)	8 (5 − 14)	9 (5 − 17)
CD4^+^ T lymphocytes				
CD4^+^ (x10^9^/l)	2.34 (1.11 − 3.03)	1.3 (0.76 − 2.33)	1.15 (0.66 − 1.98)	0.89 (0.65 − 1.27)
CD4^+^ (%)^b^	65 (55 − 71)	60 (51 − 74)	59 (50 − 63)	60 (48 − 65)
Naive CD4^+^ (x10^9^/l)	2 (0.88 − 2.74)	1.01 (0.56 − 1.88)	0.92 (0.46 − 1.61)	0.65 (0.45 − 0.87)
Naive CD4^+^ (%)^c^	86 (82 − 92)	80 (75 − 85)	79 (64 − 83)	70 (60 − 75)
Recent thymic emigrant, RTE (x10^9^/l)	1.91 (0.47 − 2.69)	0.86 (0.45 − 1.6)	0.81 (0.39 − 1.38)	0.47 (0.35 − 0.71)
RTE (%)^c^	74 (62 − 83)	66 (56 − 70)	66 (55 − 70)	56 (50 − 61)
CD4^+^CD45RA^+^ CD62L^+^ (%)^c^	87 (82 − 91)	78 (70 − 84)	79 (65 − 81)	68 (59 − 73)
CD4^+^ TCM, central memory (%)^c^	9 (6 − 16)	16 (11 − 20)	15 (13 − 24)	21 (17 − 30)
CD4^+^ TEM, effector memory (%)^c^	3 (2 − 5)	4 (3 − 6)	6 (4 − 13)	8 (5 − 11)
CD4^+^ TEMRA, terminally differentiated effector (%)^c^	0.9 (0.3 − 1.6)	0.6 (0.2 − 0.8)	0.6 (0.4 − 1)	0.6 (0.3 − 1.1)
HLA-DR^+^ CD38^+^CD4^+^ activated T helper (%)^c^	3 (2 − 4)	3 (2 − 5)	3 (2 − 4)	3 (2 − 4)
Regulatory CD4^+^ T, Treg (%)^c^	6 (5 − 10)	7 (5 − 9)	8 (7 − 10)	7 (5 − 10)
CD8^+^ cytotoxic T lymphocytes				
CD8^+^ (x10^9^/l)	1.06 (0.59 − 1.69)	0.54 (0.28 − 1.25)	0.66 (0.42 − 1.1)	0.42 (0.34 − 0.75)
CD8^+^ (%)^b^	30 (23 − 40)	33 (20 − 35)	34 (24 − 39)	30 (23 − 42)
Naive CD8^+^ (x10^9^/l)	0.77 (0.46 − 0.99)	0.44 (0.22 − 0.77)	0.45 (0.25 − 0.67)	0.3 (0.19 − 0.42)
Naive CD8^+^ (%)^d^	79 (53 − 88)	73 (60 − 86)	68 (55 − 77)	58 (46 − 77)
CD8^+^ TCM (%)^d^	2 (1 − 3)	3 (1 − 4)	2 (1 − 2)	2 (1 − 5)
CD8^+^ TEM (%)^d^	6 (5 − 14)	11 (5 − 17)	13 (5 − 22)	13 (6 − 27)
CD8^+^ TEMRA (%)^d^	12 (5 − 26)	10 (6 − 26)	18 (13 − 27)	18 (9 − 34)
HLA-DR^+^ CD38^+^CD8^+^ activated cytotoxic T (%)^d^	5 (2 − 15)	7 (3 − 21)	8 (4 − 12)	6 (3 − 15)
TCRαβ^+^ double-negative T, DNT (%)^b^	1.3 (0.7 − 2.2)	1.8 (1.1 − 2.6)	1.6 (1 − 2)	1.7 (1.1 − 2.3)
Double-positive T, DPT (%)^b^	0.5 (0.3 − 0.9)	0.4 (0.2 − 0.5)	0.4 (0.2 − 0.7)	0.3 (0.2 − 0.6)

^a^ % of total lymphocytes

^b^ % of T lymphocytes (CD3^+^)

^c^ % of CD3^+^CD4^+^ lymphocytes

^d^ % of CD3^+^CD8^+^ lymphocytes

*HLA-DR* Human Leukocyte Antigen – DR isotype, *TCR* T cell receptor, *RTE* recent thymic emigrant, *TCM* central memory T cell, *TEM* effector memory T cell, *TEMRA* terminally differentiated effector T cell, *Treg* regulatory T cell, *DNT* TCRαβ + double-negative T cell, *DPT* double-positive T cell

**Table 2:** Reference values for B lymphocyte subsets and dendritic cells for children aged 0 − 12 years

	0–2 years (median: 1.1) *n* = 23	2–4 years (median: 3.4) *n* = 14	4–6 years (median: 4.9) *n* = 16	6–12 years (median: 8.1) *n* = 15
Naive B lymphocytes (%)	83 (75 − 89)	73 (66 − 79)	66 (51 − 74)	56 (45 − 66)
Memory B lymphocytes (%)	13 (9 − 23)	25 (17 − 32)	29 (23 − 45)	41 (29 − 53)
Non-switched memory B, nsmB (%)	10 (7 − 15)	14 (9 − 21)	15 (11 − 23)	20 (12 − 27)
Switched memory B, smB (%)	2 (1 − 3)	5 (4 − 10)	10 (6 − 24)	14 (8 − 21)
CD21_low_CD38_low_ activated B (%)	6 (3 − 8)	8 (5 − 11)	10 (6 − 13)	10 (6 − 20)
CD19^+^CD38_high_IgM_high_ transitional B (%)	12 (8 − 17)	9 (7 − 15)	6 (3 − 11)	4 (3 − 6)
Plasmablasts (%)	0.6 (0.2 − 1.4)	0.3 (0.2 − 2.0)	1 (0.3 − 2.8)	0.6 (0.2 − 1.6)
Plasmacytoid dendritic cells, pDC (%)	0.18 (0.06 − 0.32)	0.18 (0.08 − 0.38)	0.2 (0.09 − 0.37)	0.18 (0.11 − 0.48)
Myeloid dendritic cells, mDC (%)	0.08 (0.02 − 0.16)	0.08 (0.02 − 0.18)	0.15 (0.07 − 0.29)	0.1 (0.03 − 0.2)

Percentages are shown as median and percentile range (P10 − P90). B cell subsets are shown as % of CD19+ B cells, and dendritic cells as % of peripheral blood leucocytes

### Reference Value Studies Present Considerable Variability in Statistical and Methodological Approaches

We conducted a literature review on studies that have previously assessed pediatric reference values for extended lymphocyte immunophenotyping with the aim of examining how the values of diagnostically essential T and B lymphocyte populations obtained in this study differ from previous results. Naive CD4^+^ and CD8^+^ T cells, RTEs, DNTs, CD8^+^TEMRAs and γδ T cells were included from the T cell compartment and naive, total memory, switched memory, non-switched memory, transitional, activated B cells and plasmablasts from the B cell compartment. Articles chosen for the literature review were acquired in July 2024 from PubMed using following search terms: pediatric, T lymphocyte/cell, B lymphocyte/cell, subpopulation/subset and reference values. Additional literature searches were performed for more specified cell subpopulations, such as RTEs and DNTs. Articles that presented their reference values in the main article or supplementary files were considered, and articles that provided reference values only as a chart were excluded. We did not inquire the raw data from the authors.

**Fig. 2:**
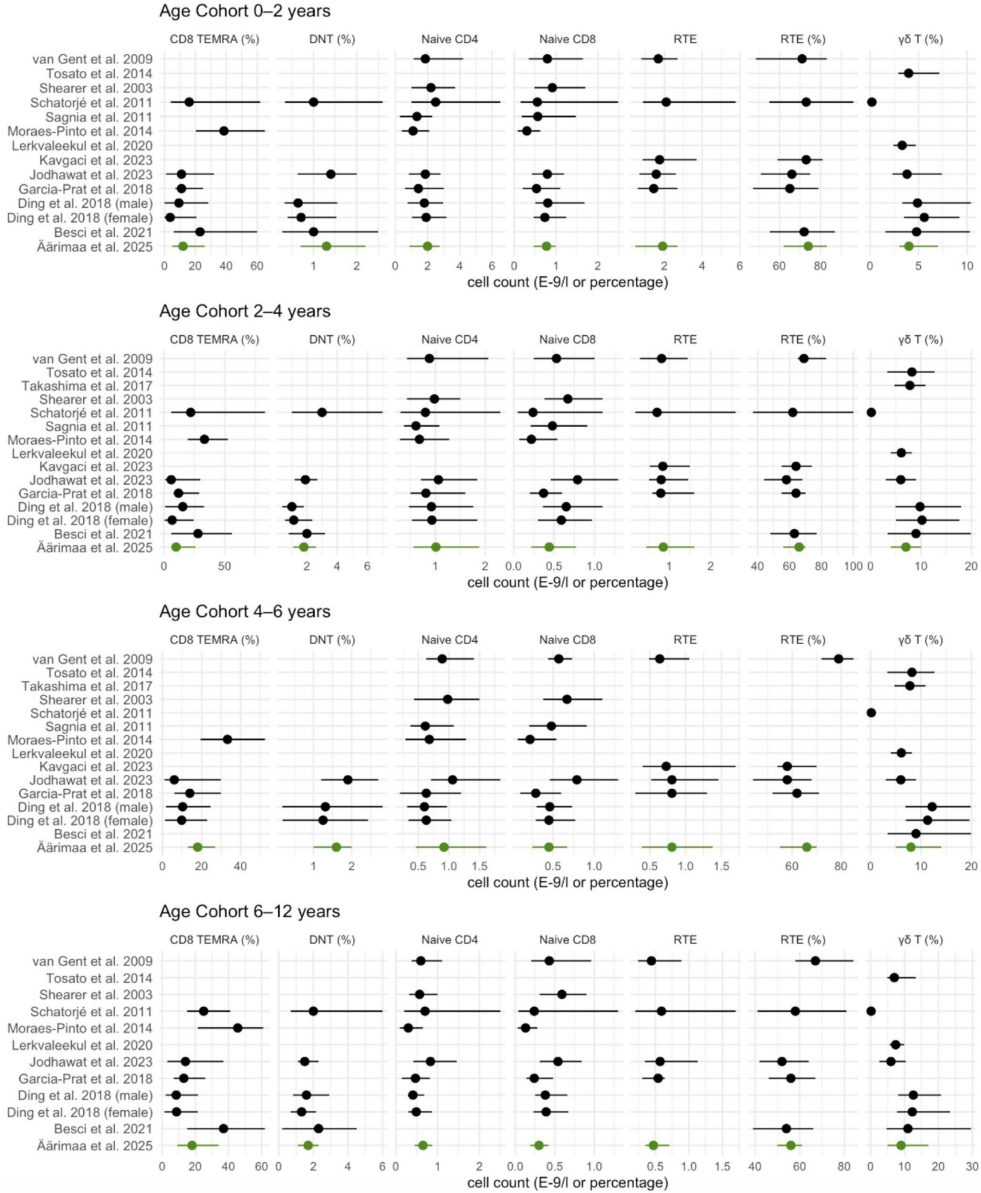
Comparison of reference values for key T cell subsets in previous studies by age. The results from the present study are showcased in green font

**Fig. 3:**
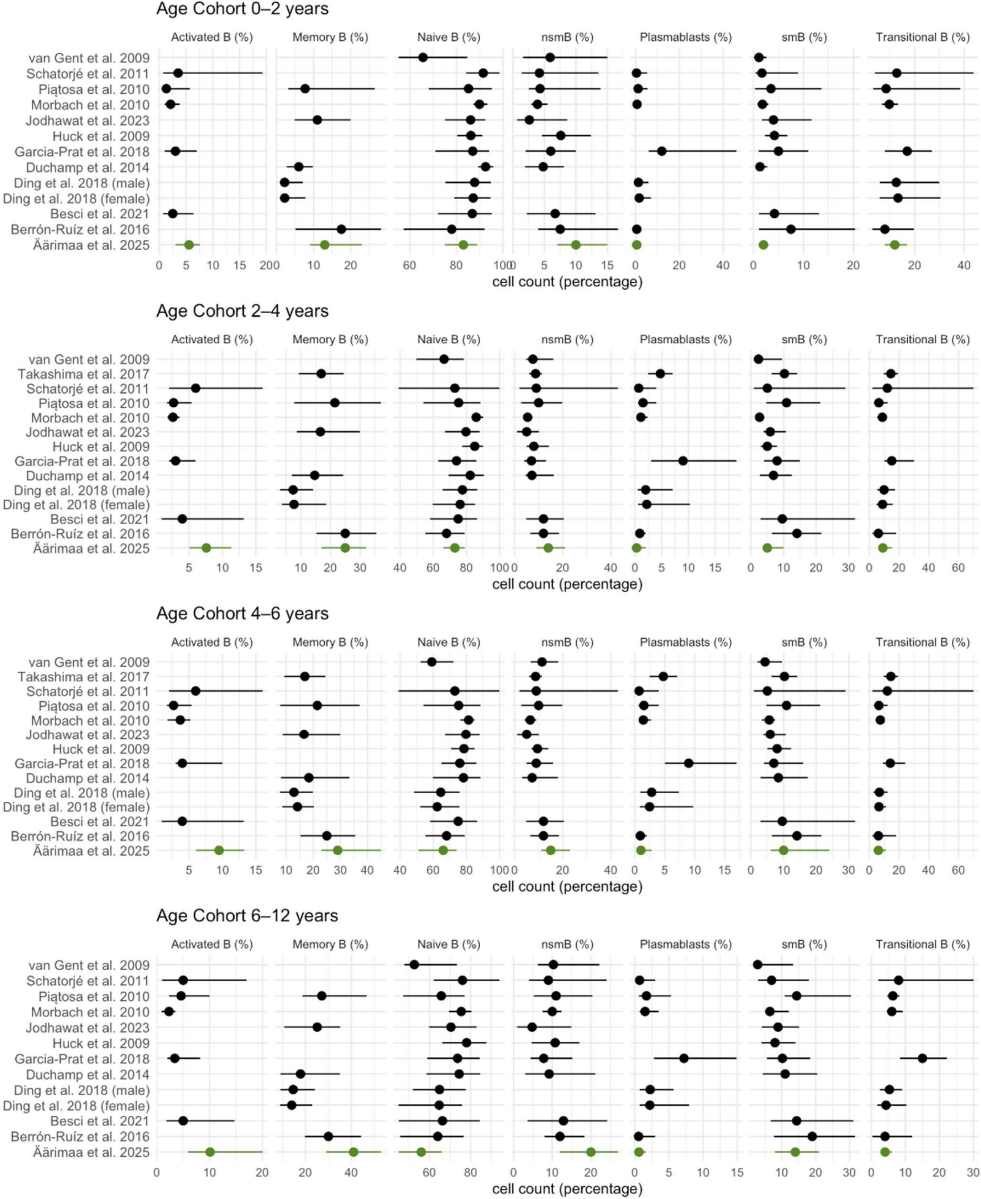
Comparison of reference values for key B cell subsets in previous studies by age. The results from the present study are showcased in green font

The literature search resulted in 13 studies that have at least partly assessed the included T cell subpopulations [5, 6, 8, 12–21] and 12 studies [4, 6, 8, 17, 19–26] for the B cell subpopulations in the pediatric cohorts. Six studies assessed both T and B cell subsets [6, 8, 17, 19–21], and overall, 19 studies were included in the analyses [4–6, 8, 12–26]. One study evaluated the reference values for male and female separately and for the comparison, we included both values [21]. The results of the comparison analysis are shown in Figs. 2 and 3, and the articles, their chosen statistical interval, age cohorts and cell subset definitions are provided in detail in Supplementary Tables S3 and S4 as well as details on how the age cohorts, which varied significantly, were compared between the studies.

The chosen statistical interval, age cohorts and cell subset definitions varied between the studies. Fourteen studies used percentile range, which varied overall from 5 − 95 to 25 − 75 percentiles [4, 6, 12–18, 21–25]. Three studies chose tolerance interval with the included proportion being 90% (0.90) and confidence level of 0.95 [5, 19, 26]. One study reported value range of highest and lowest value present in their age-matched cohorts [8], and one study used standard deviation of the population as the statistical interval for both T and B cell compartments [20]. The comparison shows that the largest differences in the values seems to result from differences in the chosen interval, with tolerance intervals providing especially wide reference limits [5, 19, 26].

Cell subset definitions varied slightly between the studies with most differences in RTE and DNT cell definition (Supplementary Table S3). RTEs were defined as either CD45RA^+^CD31^+^, CD45RA^+^CD62L^+^CD31^+^, or CD45RA^+^CD27^+^CD62L^+^CD31^+^, however the reference values from these definitions were similar. In two studies, it was not unequivocally stated whether DNTs also included TCRγδ^+^ cells [15, 19]. Transitional B cells were defined in most cases as either CD19^+^CD38^high^IgM^high^ and CD19^+^CD24^high^CD38^high^. Garcia-Prat et al. [6] defined transitional B cells as CD19^+^CD27^–^CD24^+^CD38^+^IgD^+^ and their provided median seems to be slightly higher compared to other studies. Their study also defines plasmablasts as CD19^+^CD27^+^CD24^–^CD38^+^IgD^–^ with higher median and wider reference limits. Otherwise, plasmablasts were either defined as CD19^+^CD24^–^CD38^high^, CD19^+^CD38^high^IgM^–^, or CD19^+^CD24^–^CD38^high^IgM^–^.

Our reference intervals for non-switched memory and activated B cells are moderately higher compared to previous studies. However, the overall results of this study and previous studies seem to comply with each other relatively well and while the reference limits do differ between some of the studies, the obtained median remains rather similar in all studies and in every age cohort.

### γδ T Cells are Increased in CHH while Naïve T Cells are Decreased in Most IEIs

**Fig. 4:**
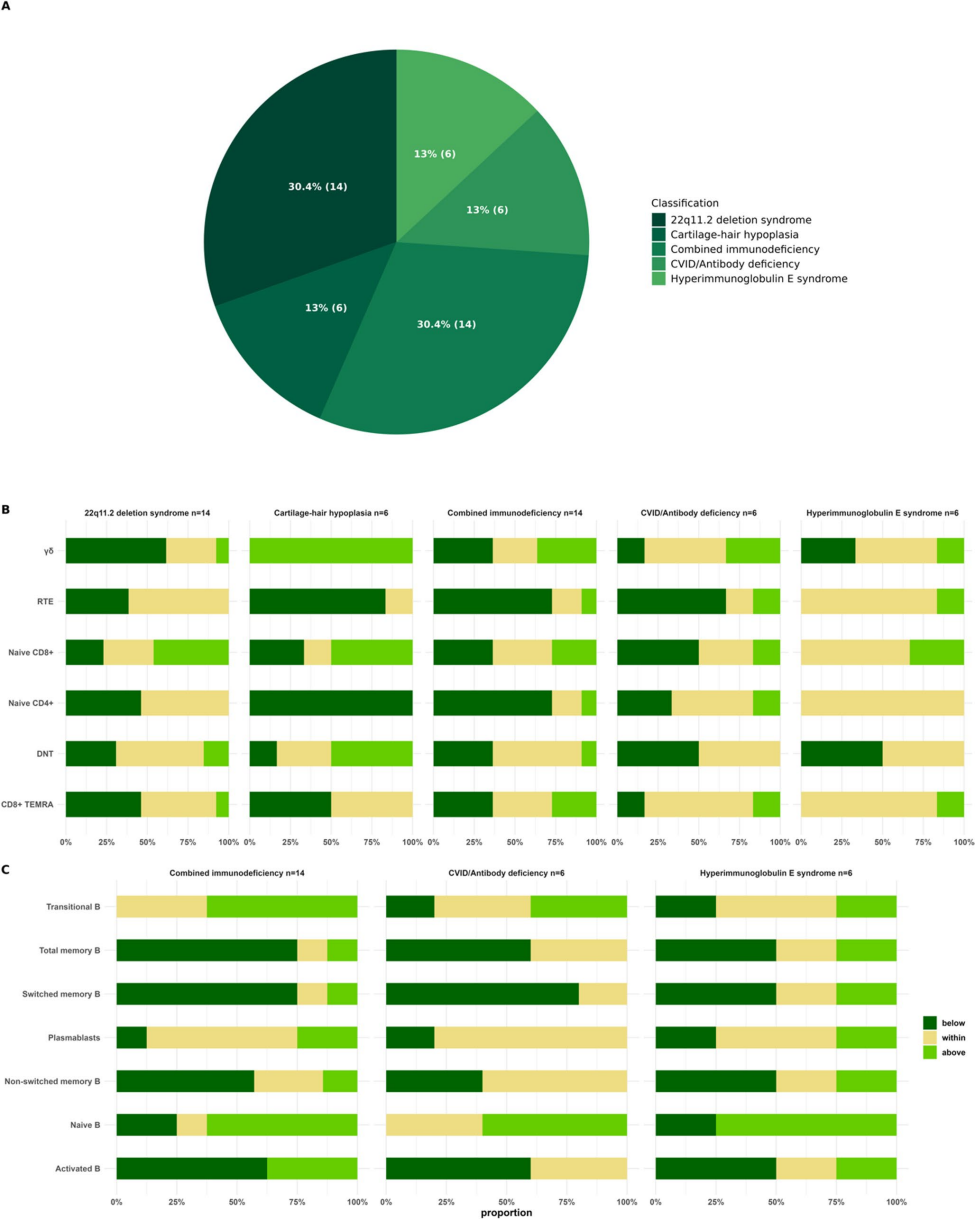
Results from the pediatric IEI cohorts. All patients did not have immunophenotyping data for all cell subsets and thus the count (n) provided in Fig. 4B and 4C constitutes the size of the specific IEI cohort, not the number of samples available for that cell subset. Detailed information on how many patients had specific cell subset data available is provided in Supplementary Tables S5 and S6. (**A**) Classification of IEI cohorts based on the IEI diagnosis. (**B**) Key T cell subset values of IEI cohorts compared to reference values. (**C**) B cell subset values of IEI cohorts compared to reference values

Figure 4 shows the diagnoses and their proportions in the IEI cohort (Fig. 4A), and the proportions of patient sample values below, within and above the assessed reference values (Fig. 4B and C). The most common diagnoses in the clinical cohort were 22q11.2del and CID (both *n* = 14, 30.4%). The proportional values of RTEs (38%), naïve CD4^+^ (46%), CD8^+^TEMRAs (46%), and γδ T cells (62%) were below the reference range, while naïve CD8 + T cells were above the reference range (46%) in the 22q11.2del cohort. In cartilage-hair hypoplasia (CHH; *n* = 6, 13%), DNTs (50%), naïve CD8+ (50%), and γδ T cells (100%) were above, while RTEs (83%) and naïve CD4 + T cells (100%) below the reference range. Values of naïve CD4+ (73%) and RTEs (73%) were below the reference range in CID cohort. CVID or antibody deficiency cohort also had RTEs (67%) below the reference range as well as naïve CD8 + T (50%) and DNT cells (50%). Patients with HIES had T lymphocyte values mostly within the reference range (Fig. 4B).

### Patients with CID and CVID have Increased Naïve and Transitional B and Decreased Memory B Cells

Levels of total memory, switched and non-switched memory, and activated B cells followed similar pattern together and were below the reference ranges in CVID or antibody deficiency, CID, and HIES cohorts (Fig. 4C). Naïve B cells were above the reference range in CID (63%), CVID or antibody deficiency (60%), and HIES (75%) cohorts. Transitional B cells were above the reference range in CID (63%) and CVID or antibody deficiency (40%) cohorts. Plasmablasts were mostly within the reference range in all IEI cohorts.

### Children have Weaker Lymphocyte Stimulation Responses than Adults

**Fig. 5:**
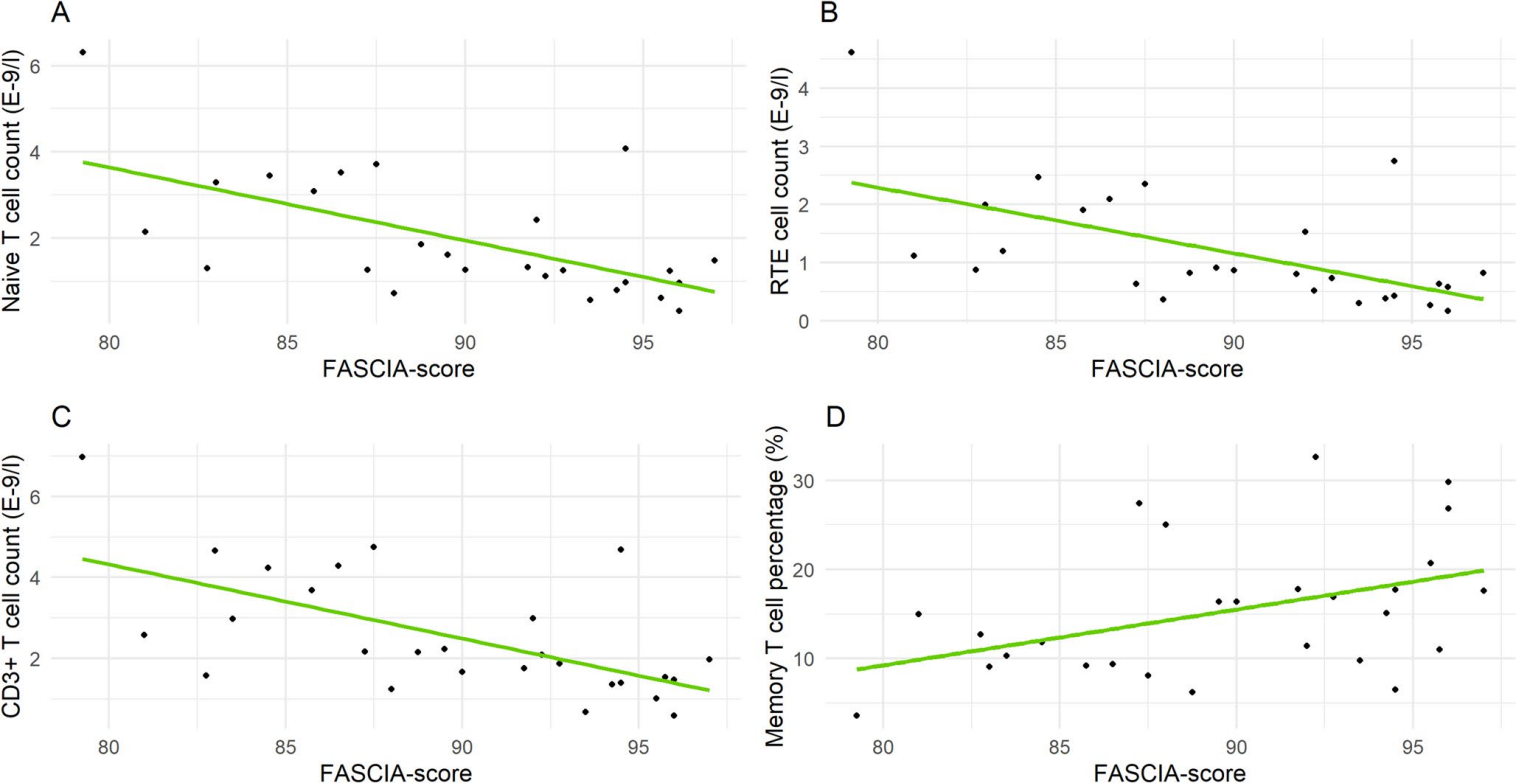
Correlations between FASCIA score and different lymphocyte subset counts. (**A**) Correlation between FASCIA-score and naïve T cell count. (**B**) Correlation between FASCIA-score and RTE cell count. (**C**) Correlation between FASCIA-score and CD3^+^ T cell count. (**D**) Correlation between FASCIA-score and memory T cell count

The lower reference limits for FASCIA from healthy children were significantly lower than those for adults (Table 3) and this was evident with both mitogens used in the stimulation (PHA, ConA). To evaluate this difference further, we tested the correlation of combined FASCIA-score and the level of naive and memory T lymphocytes. The FASCIA-score represents the average percentage of blasts among CD4 + and CD8 + T-lymphocytes in response to both mitogens (PHA, ConA), providing an estimate of the cells overall responsiveness. A previous study by Lusila et al. [27], which was conducted on a heterogeneous population consisting mainly of adults examined for clinical suspicion of IEI, found no correlation between FASCIA-score and absolute total T lymphocyte count (Spearman’s rank correlation coefficient − 0.043). In contrast, our analysis demonstrated strong negative correlations between FASCIA-score and absolute naive T cell count (− 0.604, Table 3) and absolute RTE count (− 0.635). Additionally, we found a strong negative correlation with FASCIA-score and absolute total T lymphocyte count (− 0.610). A moderate positive correlation was found between FASCIA-score and memory T cell percentage (0.460). Correlation plots are shown in Fig. 5.

**Table 3:** Lower reference limits for lymphocyte mitogen stimulation with FASCIA method for children under 9 years of age and adults and the results from the spearman’s correlation analysis of combined FASCIA-score and T lymphocyte subpopulations. Correlation analysis was performed only for pediatric samples

Stimulation	Children (*n* = 27)	Adults (*n* = 177)
CD4 + PHA	88%	92%
CD8 + PHA	82%	87%
CD4 + ConA	71%	77%
CD8 + ConA	67%	76%
*Lymphocyte population (absolute counts)*	*Spearman´s correlation coefficient*	
Total T cells	−0.610	
Naïve T cells	−0.604	
RTE cells	−0.635	
Memory T cells	0.460	

*FASCIA* flow-cytometric assay for specific cell-mediated immune-response in activated whole blood, *PHA* phytohemagglutinin, *ConA* Concavalin A, *RTE* recent thymic emigrant

## Discussion

In this study, we established age-matched reference values for T and B lymphocyte subsets and dendritic cells for children aged 0 − 12 years and compared our results of key lymphocyte subsets to reference values obtained from studies around the world. To our knowledge, only Jodhawat et al. [17] has compared the earlier work of pediatric reference values of extended immunophenotyping, albeit their comparison solely focused on T-cell subsets and did not include all published literature. The changes in the lymphocyte compartment underwent similar age-associated developments as evident in the previous publications. The methodology of reference value assessment differed between the studies. We compared our reference values with values from children with IEI and found values of γδ T cells above the reference range in all CHH patients and RTEs below in CHH, CID and CVID. Additionally, we assessed suggestive reference limits for lymphocyte stimulation test (FASCIA) in children and compared their results with adults. We found weaker stimulation results in children than adults.

Commonly used upper reference limits for DNTs in ALPS diagnostics are 2% or 2.5% of CD3^+^ T cells [9, 28]. In our study, the upper reference limits of all age groups exceeded 2%, with the highest upper reference limit being 2.9% in children aged 6 − 12 years. Results higher than 2% were also present in all studies included in the literature comparison of DNTs, however, the medians of our age cohorts as well as most other studies remained below the 2% limit. Overall, our findings suggest that healthy children may naturally have slightly higher DNTs than the commonly used 2% of CD3^+^ T cells, although the specific DNT cutoff levels are laboratory-dependent and cannot be generalized to all laboratories by default.

Earlier studies have suggested the importance of country-specific reference values as the environment and the prevalence of infectious diseases have been considered to affect the development of the lymphocyte compartment [29]. However, Shearer et al. found that the effect of ethnicity and sex appeared small. In their study, inter-laboratory variability was as significant a confounder as the age of the participant [13]. Although we did not utilize meta-analytic methods, our analysis approach did not find major differences between the studies conducted around the world. The median values of key lymphocyte subpopulations remained similar, and most differences occurred in the width of the reference interval. These discrepancies seem to result from differences in laboratory or statistical methodologies. The cell-surface markers used for extended immunophenotyping varied among the studies as the field continues to lack proper standardization among lymphocyte definitions. A notable difference concerns the various definitions of naïve T cells. While our laboratory defines naïve T cells using both CD45RA and CCR7 expression, the custom in many laboratories is to rely on CD45RA alone, which contributes to variability [5, 6, 14, 17].

The largest differences in reference intervals were attributed to the chosen statistical method. Reference intervals by Schatorjé et al. [5, 26] and Besci et al. [19] were in almost all cases wider than in other studies. This seems to be the result of using tolerance intervals as reference range. Discussion about the most appropriate method for reference range estimation has been published recently [30, 31]. We chose percentile-based reference intervals as tolerance intervals inherently produce broader intervals and as such, in medicine, decrease the risk for false positives but increases the risk for false negative results in clinical practice. Other studies have used alternative percentile-based approaches – such as P25 − P75 (IQR), P10 − P90, or P5 − P95 intervals – with each reflecting different priorities in balancing diagnostic sensitivity and specificity [6, 15, 16]. The lack of consensus highlights the challenge of defining universally applicable reference intervals, especially in pediatric populations where the cohorts need to be age-matched as the immune system undergoes constant development. Additionally, as the reference intervals were wide in our literature review across the studies, it is evident that the biological variation of lymphocyte subsets among healthy children is significant. While the risk of overdiagnosis may be present when narrower reference ranges are used, we believe that the more sensitive detection of immunologically vulnerable individuals outweighs this risk. Furthermore, as extended lymphocyte immunophenotyping is only ordered by a specialized immunologist who not only accounts for laboratory results but also evaluates the patient’s clinical history and pathognomonic symptom occurrence [32], we argue that the real risk of overdiagnosis due to a narrow reference range remains small.

In 22q11.2del cohort, the proportional values of naïve T cells were not below the reference range for most patients. Although characterized as an immunodeficiency syndrome, the immunophenotypes of 22q11.2del vary greatly and not all patients suffer from severe thymic dysfunction nor have naïve T cell lymphopenia. Complete DiGeorge syndrome (cDGS) characterized by athymia and total T cell lymphopenia is extremely rare [33, 34]. Accumulated DNTs have been reported in DGS patients with autoimmune cytopenia or ALPS-like features [35, 36]. In our cohort, DNTs were above the reference range only for a minority of patients.

The contribution of patients with cartilage-hair-hypoplasia (CHH) is relatively large (*n* = 6) compared to the overall prevalence of this condition in the world. CHH is of the Finnish Disease Heritage, i.e. group of rare genetic diseases enriched in the Finnish population due to a founder effect. All peripheral γδ T cell values of CHH patients were above the reference range. DNTs were also above the reference range for half of the CHH patients. Patients with CHH have an increased risk of developing autoimmune diseases, severe infections, and cancer [37]. Previous findings of decreased naïve CD4^+^ cells and RTEs in CHH were also evident in this study [38, 39]. Our findings of increased γδ T cells and DNTs could partly explain how immune dysregulation in CHH emerges as γδ T cells can have autoimmune effects [40]. However, as our CHH cohort only included 6 patients, these findings remain preliminary. Naïve CD8^+^ T cells were increased in most patients with CHH, but this is likely due to the especially low level naïve CD4^+^ cells which increases the proportional size of the CD8 + cells in the naïve cell compartment. In our CID and CVID cohorts, 33 − 36% had γδ T cell values above and 17 − 27% had CD8^+^ TEMRAs above the reference range. Human cytomegalovirus (HCMV) infection has been shown to increase the levels of γδ T cells and drive T lymphocyte differentiation towards terminal stage, especially in CID and CVID patients [41–43]. We did not have data on HCMV status of any study participants nor the absolute lymphocyte values of IEI patients.

Due to the variabilities in the laboratory practices of lymphocyte stimulation, our reference values for FASCIA are not generalizable to other laboratories. However, as the reference limits were assessed similarly as with the adult population, we were able to compare the results between children and adults. Children tend to have modestly weaker stimulation responses in FASCIA than adults which is further emphasized by the fact that the children’s reference limit was 10th and adults’ 5th percentile of the distribution. This difference could be explained by children having proportionally more naive lymphocytes whereas adults have more memory lymphocytes. Supporting this hypothesis, we found strong negative correlations between FASCIA-score and absolute naive T, RTE and total T cell counts and moderate positive correlation with the T memory cell percentage in children. One plausible biological explanation for the lower proliferative rate for naïve T cells in FASCIA lymphocyte stimulation could stem from the long stimulation period used in FASCIA. Contrary to memory cells, naïve T cells lose their proliferative capacity in longer mitogen stimulation, which is used in FASCIA method unlike other lymphocyte stimulation tests [44]. Our findings suggest that children and adults have differing results in lymphocyte stimulation, and this is potentially driven by the higher proportion of naïve T cells in children.

Our study has some limitations, mainly the limited sample size, particularly in children under one year of age. This prevented further subdivision of age cohorts. Changes in the lymphocyte compartment start to occur quickly after birth and the lymphocyte composition of a 2-year-old differs from an infant [7, 8]. Most significant change after birth is the heavily decreasing RTE count [7]. Our quantile regression model for RTE count reflects this exponential decrease after 1 year of age. A robust review of the reference value literature was conducted to improve the reliability of our reference values. However, as the studies included in this comparison used varying age grouping – particularly in the younger cohorts – we combined these age groups together to include them in our comparison. This can be seen as a limitation as it might result in slightly different reference limits than the original data would have provided. Even though the IEI cohorts included patients with rare diseases and thus assembling an ample cohort is challenging, sufficient sample size remains essential when generalizing results to a specific condition. As the sample size is limited, the IEI analysis provides a depiction of how these cell subsets possibly behave in IEI-affected children, and does not provide a clinical validation of our reference ranges. We did not collect information about the participants’ ethnicity, albeit in our estimation the majority of the study participants are of Finnish ethnicity. This homogeneity limits the generalizability of our results. However, reference values for pediatric extended immunophenotyping have not been published previously in Northern Europe. Our reference ranges cannot be directly extended to all laboratories but are applicable to the ones using comparable methodology and analytical platforms.

## Conclusions

In conclusion, this study provides a considerable contribution with establishing reference values for T and B lymphocyte subpopulations and dendritic cells for children aged 0 to 12 years. Our reference values were compared with both earlier scientific work and patient samples. The elevation of γδ T cells and DNTs in many IEI conditions needs more research as these cell populations constitute interesting biomarker candidates. Additionally, we established reference limits for lymphocyte stimulation with FASCIA in adults and suggestive reference limits for children under 9 years of age and found a correlation with weaker mitogen response and the level of naive T lymphocytes in children. Lymphocyte immunophenotyping lacks universal standardization of lymphocyte subsets which complicate the comparison between laboratories. Here, our immunophenotypes have been selected to reflect the most accepted definitions of different populations. Moreover, our comparison to previous literature confirmed that our reference values are in good agreement with other published reference values. While IEIs remain rather rare medical conditions overall, the prevalence and the number of people affected by them are steadily increasing and thus the improvement of laboratory diagnostics of these conditions is essential. We hope this work aids clinicians in interpreting lymphocyte immunophenotypes in immunologically affected children more accurately and precipitates the diagnostic process for immune-mediated conditions in pediatrics.

## Supplementary Information

Below is the link to the electronic supplementary material.

Supplementary Material 1 (DOCX 1.87 MB)

## Data Availability

The datasets of this study are available from the corresponding author (E.Ä.) upon reasonable request.

## Abbreviations

22q11.2del: 22q11.2 deletion syndrome (DiGeorge syndrome, DGS)
ALPS: autoimmune lymphoproliferative syndrome
BD: BD Biosciences (Becton Dickinson)
BSA: bovine serum albumin
CD: cluster of differentiation
CHH: cartilage-hair hypoplasia
CI: confidence interval
CID: combined immunodeficiency
ConA: Concavalin A
DNT: double-negative TCRαβ⁺ T cell
DPT: double-positive T cell
EDTA: ethylenediaminetetraacetic acid
FASCIA: flow-cytometric assay for specific cell-mediated immune-response in activated whole blood
HCMV: human cytomegalovirus
HIES: hyperimmunoglobulin E syndrome
HSCT: hematopoietic stem cell transplantation
HUS: Hospital District of Helsinki and Uusimaa
ICD-10: 10th revision of the International Statistical Classification of Diseases and Related Health Problems
IEI: inborn errors of immunity
IQR: interquartile range
mDC: myeloid dendritic cell
MRI: magnetic resonance imaging
nsmB: non-switched memory B cell
PBS: phosphate-buffered saline
pDC: plasmacytoid dendritic cell
PHA: phytohemagglutinin
PR: percentile range
RTE: recent thymic emigrant
SD: standard deviation
smB: switched memory B cell
TCM: central memory T cell
TCR: T cell receptor
TEM: effector memory T cell
TEMRA: terminally differentiated effector T cell
TI: tolerance interval
Treg: regulatory CD4+ T cell

## References

[1] Neirinck J, Emmaneel A, Buysse M, Philippé J, Van Gassen S, Saeys Y, et al. The euroflow PID orientation tube in the diagnostic workup of primary immunodeficiency: daily practice performance in a tertiary university hospital. Front Immunol. 2022;13:937738. 36177024 10.3389/fimmu.2022.937738 PMC9513319

[2] Oliveira JB, Fleisher TA. Molecular- and flow cytometry-based diagnosis of primary immunodeficiency disorders. Curr Allergy Asthma Rep. 2010;10(6):460–7. 20683683 10.1007/s11882-010-0137-8

[3] Kanegane H, Hoshino A, Okano T, Yasumi T, Wada T, Takada H, et al. Flow cytometry-based diagnosis of primary immunodeficiency diseases. Allergol Int. 2018;67(1):43–54. 28684198 10.1016/j.alit.2017.06.003

[4] Morbach H, Eichhorn EM, Liese JG, Girschick HJ. Reference values for B cell subpopulations from infancy to adulthood. Clin Exp Immunol. 2010;162(2):271–9. 20854328 10.1111/j.1365-2249.2010.04206.x PMC2996594

[5] Schatorjé EJH, Gemen EFA, Driessen GJA, Leuvenink J, van Hout RWNM, de Vries E. Paediatric reference values for the peripheral T cell compartment. Scand J Immunol. 2012;75(4):436–44. 22420532 10.1111/j.1365-3083.2012.02671.x

[6] Garcia-Prat M, Álvarez-Sierra D, Aguiló-Cucurull A, Salgado-Perandrés S, Briongos-Sebastian S, Franco-Jarava C, et al. Extended immunophenotyping reference values in a healthy pediatric population. Cytometry B Clin Cytom. 2019;96(3):223–33. 30334372 10.1002/cyto.b.21728

[7] Drozdov D, Petermann K, Dougoud S, Oberholzer S, Held L, Güngör T, et al. Dynamics of recent thymic emigrants in pediatric recipients of allogeneic hematopoetic stem cell transplantation. Bone Marrow Transplant. 2022;57(4):620–6. 35140350 10.1038/s41409-022-01594-w

[8] van Gent R, van Tilburg CM, Nibbelke EE, Otto SA, Gaiser JF, Janssens-Korpela PL, et al. Refined characterization and reference values of the pediatric T- and B-cell compartments. Clin Immunol. 2009;133(1):95–107. 19586803 10.1016/j.clim.2009.05.020

[9] Oliveira JB, Bleesing JJ, Dianzani U, Fleisher TA, Jaffe ES, Lenardo MJ et al. Revised diagnostic criteria and classification for the autoimmune lymphoproliferative syndrome (ALPS): report from the 2009 NIH International Workshop. Blood. 2010;116(14):e35–40. 20538792 10.1182/blood-2010-04-280347 PMC2953894

[10] Seidel MG, Kindle G, Gathmann B, Quinti I, Buckland M, van Montfrans J, et al. The European society for immunodeficiencies (ESID) registry working definitions for the clinical diagnosis of inborn errors of immunity. The Journal of Allergy and Clinical Immunology: In Practice. 2019;7(6):1763–70. 30776527 10.1016/j.jaip.2019.02.004

[11] R Core Team. (2022). R: A language and environment for statistical computing. R Foundation for Statistical Computing, Vienna, Austria. URL https://www.R-project.org/

[12] Tosato F, Bucciol G, Pantano G, Putti MC, Sanzari M, Basso G, et al. Lymphocytes subsets reference values in childhood. Cytometry Part A. 2015;87(1):81–5. 10.1002/cyto.a.22520 25132325

[13] Shearer WT, Rosenblatt HM, Gelman RS, Oyomopito R, Plaeger S, Stiehm ER, et al. Lymphocyte subsets in healthy children from birth through 18 years of age: the pediatric AIDS clinical trials group P1009 study. J Allergy Clin Immunol. 2003;112(5):973–80. 14610491 10.1016/j.jaci.2003.07.003

[14] de Moraes-Pinto MI, Santos-Valente OE, Almeida EC, Andrade LC, de Dinelli PR. Lymphocyte subsets in human immunodeficiency virus-unexposed Brazilian individuals from birth to adulthood. Mem Inst Oswaldo Cruz. 2014;109(8):989–98. 25424448 10.1590/0074-0276140182 PMC4325616

[15] Lerkvaleekul B, Apiwattanakul N, Klinmalai C, Hongeng S, Vilaiyuk S. Age-related changes in lymphocyte subpopulations in healthy Thai children. J Clin Lab Anal. 2020;34(5):e23156. 31855295 10.1002/jcla.23156 PMC7246386

[16] Kavgacı A, Bayrakoğlu D, Bal SK, Haskoloğlu Ş, Çullas-İlarslan NE, Topçu S, et al. Evaluation of thymopoiesis in healthy Turkish children aged 0–6 years. Turk J Pediatr. 2023;65(1):73–80. 36866987 10.24953/turkjped.2021.5190

[17] Jodhawat N, Bargir UA, Setia P, Taur P, Bala N, Madkaikar A, et al. Normative data for paediatric lymphocyte subsets: a pilot study from Western India. Indian J Med Res. 2023;158(2):161–74. 37787259 10.4103/ijmr.ijmr_3282_21 PMC10645029

[18] Sagnia B, Ndongo FA, Tetang SNM, Torimiro JN, Cairo C, Domkam I, et al. Reference values of lymphocyte subsets in healthy, HIV-negative children in Cameroon. Clin Vaccine Immunol. 2011;18(5):790. 21411603 10.1128/CVI.00483-10 PMC3122514

[19] Besci Ö, Başer D, Öğülür İ, Berberoğlu AC, Kıykım A, Besci T, et al. Reference values for T and B lymphocyte subpopulations in Turkish children and adults. Turk J Med Sci. 2021;51(4):1814–24. 33754649 10.3906/sag-2010-176 PMC8569764

[20] Takashima T, Okamura M, Yeh T wen, Okano T, Yamashita M, Tanaka K, et al. Multicolor flow cytometry for the diagnosis of primary immunodeficiency diseases. J Clin Immunol. 2017;37(5):486–95. 28597144 10.1007/s10875-017-0405-7

[21] Ding Y, Zhou L, Xia Y, Wang W, Wang Y, Li L, et al. Reference values for peripheral blood lymphocyte subsets of healthy children in China. J Allergy Clin Immunol. 2018;142(3):970–e9738. 29746882 10.1016/j.jaci.2018.04.022

[22] Piątosa B, Wolska-Kuśnierz B, Pac M, Siewiera K, Gałkowska E, Bernatowska E. B cell subsets in healthy children: reference values for evaluation of B cell maturation process in peripheral blood. Cytometry B Clin Cytom. 2010;78(6):372–81. 20533385 10.1002/cyto.b.20536

[23] Huck K, Feyen O, Ghosh S, Beltz K, Bellert S, Niehues T. Memory B-cells in healthy and antibody-deficient children. Clin Immunol. 2009;131(1):50–9. 19162556 10.1016/j.clim.2008.11.008

[24] Duchamp M, Sterlin D, Diabate A, Uring-Lambert B, Guérin-El Khourouj V, Le Mauff B, et al. B-cell subpopulations in children: national reference values. Immunity Inflamm Dis. 2014;2(3):131–40. 10.1002/iid3.26 PMC4257758 25505547

[25] Berrón-Ruíz L, López-Herrera G, Ávalos-Martínez CE, Valenzuela-Ponce C, Ramírez-SanJuan E, Santoyo-Sánchez G, et al. Variations of B cell subpopulations in peripheral blood of healthy Mexican population according to age: relevance for diagnosis of primary immunodeficiencies. Allergol Immunopathol (Madr). 2016;44(6):571–9. 27780620 10.1016/j.aller.2016.05.003

[26] Schatorjé EJH, Gemen EFA, Driessen GJA, Leuvenink J, van Hout RWNM, van der Burg M, et al. Age-matched reference values for B-lymphocyte subpopulations and CVID classifications in children. Scand J Immunol. 2011;74(5):502–10. 21815909 10.1111/j.1365-3083.2011.02609.x

[27] Lusila P, Toivonen A, Jarva H, Vettenranta K, Lehtimäki S, Kekäläinen E. FASCIA method in the assessment of lymphocyte mitogen responses in the laboratory diagnostics of primary immunodeficiencies. J Clin Immunol. 2023;43(3):653–61. 36512178 10.1007/s10875-022-01417-z PMC9958160

[28] Alpha Beta Double-Negative T Cells for Autoimmune Lymphoproliferative Syndrome. Mayo Clinic Laboratories. www.mayocliniclabs.com/test-catalog/overview/82449#Clinical-and-Interpretive. Accessed 19 Dec. 2024.

[29] Mandala WL, Ananworanich J, Apornpong T, Kerr SJ, MacLennan JM, Hanson C, et al. Control lymphocyte subsets: can one country’s values serve for another’s? J Allergy Clin Immunol. 2014;134(3):759–e7618. 25171870 10.1016/j.jaci.2014.06.030 PMC4150016

[30] Liu W, Bretz F, Cortina-Borja M. Reference range: which statistical intervals to use? Stat Methods Med Res. 2021;30(2):523–34. 33054684 10.1177/0962280220961793 PMC8008401

[31] Wellek S, Jennen-Steinmetz C. Reference ranges: why tolerance intervals should not be used. Comment on Liu, Bretz and Cortina-Borja, Reference range: which statistical intervals to use? SMMR, 2021,vol. 30(2) 523–534. Stat Methods Med Res. 2022;31(11):2255–6. 35837733 10.1177/09622802221114538

[32] Abraham RS, Butte MJ. The new wholly trinity in the diagnosis and management of inborn errors of immunity. The Journal of Allergy and Clinical Immunology: In Practice. 2021;9(2):613–25. 33551037 10.1016/j.jaip.2020.11.044

[33] Davies EG. Immunodeficiency in digeorge syndrome and options for treating cases with complete athymia. Front Immunol. 2013;4:322. 24198816 10.3389/fimmu.2013.00322 PMC3814041

[34] Jyonouchi S, McDonald-McGinn DM, Bale S, Zackai EH, Sullivan KE. CHARGE syndrome and chromosome 22q11.2 deletion syndrome: a comparison of immunologic and non-immunologic phenotypic features. Pediatrics. 2009;123(5):e871–7. 19403480 10.1542/peds.2008-3400 PMC4098848

[35] Patel PK, Chinga ML, Yilmaz M, Joychan S, Ujhazi B, Ellison M, et al. Clinical and treatment history of patients with partial DiGeorge syndrome and autoimmune cytopenia at multiple centers. J Clin Immunol. 2024;44(2):42. 38231436 10.1007/s10875-023-01607-3

[36] Gu H, Mou W, Chen Z, Xie X, Yao J, Zhang R et al. Case report: Effectiveness of sirolimus in treating partial DiGeorge Syndrome with Autoimmune Lymphoproliferative Syndrome (ALPS)-like features. Front Pediatr [Internet]. 2023 Jan 18 [cited 2024 Dec 18];10. 10.3389/fped.2022.1014249 PMC9889826 36741091

[37] Vakkilainen S, Taskinen M, Mäkitie O. Immunodeficiency in cartilage-hair hypoplasia: pathogenesis, clinical course and management. Scand J Immunol. 2020;92(4):e12913. 32506568 10.1111/sji.12913

[38] de la Fuente MA, Recher M, Rider NL, Strauss KA, Morton DH, Adair M, et al. Reduced thymic output, cell cycle abnormalities, and increased apoptosis of T lymphocytes in patients with cartilage-hair hypoplasia. J Allergy Clin Immunol. 2011;128(1):139–46. 21570718 10.1016/j.jaci.2011.03.042 PMC4287238

[39] Kostjukovits S, Klemetti P, Valta H, Martelius T, Notarangelo LD, Seppänen M, et al. Analysis of clinical and Immunologic phenotype in a large cohort of children and adults with cartilage-hair hypoplasia. J Allergy Clin Immunol. 2017;140(2):612–e6145. 28284971 10.1016/j.jaci.2017.02.016 PMC5547008

[40] Shiromizu CM, Jancic CC. γδ t lymphocytes: an effector cell in autoimmunity and infection. Front Immunol. 2018;9:2389. 30386339 10.3389/fimmu.2018.02389 PMC6198062

[41] Chan S, Morgan B, Yong MK, Margetts M, Farchione AJ, Lucas EC, et al. Cytomegalovirus drives Vδ1 + γδ T cell expansion and clonality in common variable immunodeficiency. Nat Commun. 2024;15(1):4286. 38769332 10.1038/s41467-024-48527-3 PMC11106253

[42] Khairallah C, Déchanet-Merville J, Capone M. γδ T Cell-Mediated Immunity to Cytomegalovirus Infection. Front Immunol [Internet]. 2017 Feb 9 [cited 2024 Dec 18];8. 10.3389/fimmu.2017.00105 PMC5298998 28232834

[43] Klenerman P, Oxenius A. T cell responses to cytomegalovirus. Nat Rev Immunol. 2016;16(6):367–77. 27108521 10.1038/nri.2016.38

[44] Migliaccio M, Alves PMS, Romero P, Rufer N. Distinct mechanisms control human naive and antigen-experienced CD8 + T lymphocyte proliferation1. J Immunol. 2006;176(4):2173–82. 16455973 10.4049/jimmunol.176.4.2173

