# Supplementary material for: Evaluating Pediatric Reference Ranges for Extended Immunophenotyping from a Finnish Cohort against Published References
Source: J Clin Immunol. 2025 Nov 18;45(1):162. doi: 10.1007/s10875-025-01959-y (PMC12628421; doi:10.1007/s10875-025-01959-y)
Supplement: Supplementary file 1 — Supplementary Material 1 (DOCX 1.87 MB) [file 10875_2025_1959_MOESM1_ESM.pdf]

Supplementary Materials

Evaluating Pediatric Reference Ranges for Extended Immunophenotyping from a Finnish Cohort Against Published References

Journal of Clinical Immunology

Elli Äärimaa[1,2]*§, Anssi Kesäläinen[1,2]*, Saima Askeli[1,2], Anne Toivonen[2], Okko Savonius[3], Oscar Brück[4], Pauliina Lusila[1,5], Kim Vettenranta[5], Santtu Heinonen[5,6], Timo Jahnukainen[3], Minna Koskenvuo[7], Sanna Siitonen[8], Sari Lehtimäki[8], Eliisa Kekäläinen[1,2]

*These authors have contributed equally and are designated to have co-first authorship.
§Corresponding author (correspondence to: elli.aarimaa@helsinki.fi)

[1]Translational Immunology Research Program, University of Helsinki, Helsinki, Finland
[2]Clinical Microbiology, HUS Diagnostic Center, Helsinki University Hospital and University of Helsinki, Helsinki, Finland
[3]Department of Pediatric Nephrology and Transplantation, New Children's Hospital, Pediatric Research Center, Helsinki University Hospital and University of Helsinki, Helsinki, Finland
[4]Hematoscope Lab, Comprehensive Cancer Center & Department of Clinical Chemistry, Diagnostic Center, Helsinki University Hospital & University of Helsinki, Helsinki, Finland
[5]New Children's Hospital, Pediatric Research Center, Helsinki University Hospital and University of Helsinki, Helsinki, Finland
[6]FVR, Finnish Vaccine Research, Tampere, Finland
[7]Division of Pediatric Hematology, Oncology and Stem Cell Transplantation, New Children's Hospital, Helsinki University Hospital and University of Helsinki, Helsinki, Finland
[8]Clinical Chemistry, HUS Diagnostic Center, Helsinki University Hospital and University of Helsinki, Helsinki, Finland

SUPPLEMENTARY DESCRIPTION OF METHODS

Sample processing protocol, flow cytometry and cell subset definitions

Blood samples were collected in ethylenediaminetetraacetic acid (EDTA) anticoagulated vacutainer tubes. The processing of the extended immunophenotyping samples was performed within 24 hours of blood draw. For the extended immunophenotyping of T and B cells, the samples were washed three times prior to staining with phosphate-buffered saline (PBS), bovine serum albumin (BSA, 0.5%), and sodium azide NaN_3 (0.09%) to prevent nonspecific antibody binding and to remove interfering IgM antibodies from the serum in the B cell analysis. The samples for dendritic cell analysis did not undergo washing protocol.

After washing, the whole blood samples underwent incubation for 15 minutes at room temperature in the dark with a mixture of specific conjugated monoclonal antibodies tailored for each panel. Cell surface markers used to define lymphocyte and dendritic cell subpopulations and the monoclonal antibodies and fluorochromes used for the flow cytometric analysis in the HUS Diagnostic Center, Clinical Microbiology & Clinical Chemistry are provided in Supplementary Tables S1 and S2. Following the incubation, 2 ml of lysing solution was added to the samples, which were subsequently vortexed and incubated for an additional 8 minutes at room temperature in the dark. The samples were then washed with PBS + 0.5% BSA + 0.09% NaN_3 and stored at room temperature in the dark. Flow cytometry analysis was performed within 1 hour using either the FACS Canto or FACS Lyric systems (BD Biosciences), both equipped with three lasers (405 nm violet, 488 nm blue, and 647 nm red). At least 20,000 CD3+ cells were acquired for T cell analysis and 5,000 CD19+ cells for B cell analysis. For dendritic cell analysis, either 500,000 events were acquired, including all events, or until the entirety of the sample was used. The resulting data were analyzed using BD FACS Diva v9.0.1 software. Internal quality assurance procedures were implemented following the instructions from the manufacturer Becton Dickinson (BD). The gating strategies used in the extended immunophenotyping are shown in the Supplementary Figures S2, S3

and S4. Absolute values for the lymphocyte subpopulations were calculated from the absolute leukocyte and lymphocyte count obtained with Sysmex hematological analyzer (XN-2000). These absolute values were compared with values obtained from flow cytometric BD FACSLyric system with Trucount tubes (using BD Multitest reagents) (Figure S5).

Flow-cytometric assay for specific cell-mediated immune-response in activated whole blood (FASCIA) assesses the mitogenic activity of lymphocytes by cell size detection instead of radioactive markers. The overall stimulation responses of lymphocytes were assessed by the mean of percentages of activated cells per mitogen (phytohemagglutinin, PHA; Concavalin A, ConA), resulting in FASCIA score (value of 0−100). Whole blood samples were collected in heparin tubes and two primarily T cell mitogens (ConA 10 µg/ml, PHA 10 µg/ml, Sigma-Aldrich, USA) were used to stimulate diluted samples. Stimulation was performed within 27 hours of transport and storage. Samples were incubated with mitogens for seven days. Red blood cells underwent lysing (IOTest 3 Lysing Solution, Beckman Coulter, USA) and the samples were stained with conjugated antibodies (Supplementary Table S2). FACS Canto and FACS Diva software were used to obtain and analyze the flow cytometry data. FASCIA has undergone standard laboratory validation in the HUS Diagnostic Center before its clinical use. Pediatric and adult FASCIA samples underwent the same protocol.

Analysis of inter-operator variability was not performed. To minimize the inter-operator variability, the analyzers used preapplied gates with standardized analysis templates which were then adjusted by the analyzer. Over the course of the analysis, the analyzers had a possibility to consult a specialist doctor with expertise on flow-cytometric analysis.

Methodology of age grouping in the literature review

Age cohorts of articles included in the literature review differed. Of the 13 included T cell studies, 11 [1–11] had narrower subcohorts compared to the age cohort of 0−2 years old used in this study (e.g. 0−3, 3−6, 6−9, 9−12 and 12−24 months old). Similarly 10 of the 12 included B cell studies [2,7,9–16] used these narrower subcohorts. Our study included samples from 23 children aged 0 to 2 years old and with the limited sample size, we could not divide our age cohort of 0−2 years into similar subcohorts. To incorporate these studies into our comparison, we calculated the weighted average of the median and reference limits based on the number of participants in these subcohorts. If the number of participants in each subcohort was unavailable, we included the lowest value of the lower reference limit and the highest value of the higher reference limit present in the subcohorts. In these cases, the median was assessed as the lowest value present if there were less than three subcohorts and as the middlemost value when three or five subcohorts were present. Two B cell studies had age cohorts of 0−1 and 2−3 years and these subcohorts were combined with weighted average to be included in the comparison for 0−2 years [15,16]. For one study, an age cohort of 0−1 years was used for the T and B cell comparisons [11]. Similar adjustments were performed in order to match the age grouping between the studies for other age groups. Results from age cohorts of 2−5 and 2−6 years were used in the comparison of cohorts 2−4 and 4−6 years and similarly, the age cohorts of 5−10, 6−10 and 8–12 years were included in the cohort of 6−12 years for the comparison.

References

1. Schatorjé EJH, Gemen EFA, Driessen GJA, Leuvenink J, van Hout RWNM, de Vries E. Paediatric Reference Values for the Peripheral T cell Compartment. Scand J Immunol. 2012;75(4):436–44.

2. van Gent R, van Tilburg CM, Nibbelke EE, Otto SA, Gaiser JF, Janssens-Korpela PL, et al. Refined characterization and reference values of the pediatric T- and B-cell compartments. Clinical Immunology. 2009 Oct 1;133(1):95–107.

3. Tosato F, Bucciol G, Pantano G, Putti MC, Sanzari M c., Basso G, et al. Lymphocytes subsets reference values in childhood. Cytometry Part A. 2015;87(1):81–5.

4. Shearer WT, Rosenblatt HM, Gelman RS, Oyomopito R, Plaeger S, Stiehm ER, et al. Lymphocyte subsets in healthy children from birth through 18 years of age: the Pediatric AIDS Clinical Trials Group P1009 study. J Allergy Clin Immunol. 2003 Nov;112(5):973–80.

5. Moraes-Pinto MI de, Ono E, Santos-Valente EC, Almeida LC, Andrade PR de, Dinelli MIS, et al. Lymphocyte subsets in human immunodeficiency virus-unexposed Brazilian individuals from birth to adulthood. Mem Inst Oswaldo Cruz. 2014 Dec;109(8):989–98.

6. Kavgacı A, Bayrakoğlu D, Bal SK, Haskoloğlu Ş, Çullas-İlarslan NE, Topçu S, et al. Evaluation of thymopoiesis in healthy Turkish children aged 0-6 years. Turk J Pediatr. 2023;65(1):73–80.

7. Jodhawat N, Bargir UA, Setia P, Taur P, Bala N, Madkaikar A, et al. Normative data for paediatric lymphocyte subsets: A pilot study from western India. Indian J Med Res. 2023 Aug;158(2):161–74.

8. Sagnia B, Ndongo FA, Tetang SNM, Torimiro JN, Cairo C, Domkam I, et al. Reference Values of Lymphocyte Subsets in Healthy, HIV-Negative Children in Cameroon. Clinical and Vaccine Immunology : CVI. 2011 May;18(5):790.

9. Besci Ö, Başer D, Öğülür İ, Berberoğlu AC, Kıykım A, Besci T, et al. Reference values for T and B lymphocyte subpopulations in Turkish children and adults. Turk J Med Sci. 2021 Aug 30;51(4):1814–24.

10. Ding Y, Zhou L, Xia Y, Wang W, Wang Y, Li L, et al. Reference values for peripheral blood lymphocyte subsets of healthy children in China. Journal of Allergy and Clinical Immunology. 2018 Sep 1;142(3):970-973.e8.

11. Takashima T, Okamura M, Yeh T wen, Okano T, Yamashita M, Tanaka K, et al. Multicolor Flow Cytometry for the Diagnosis of Primary Immunodeficiency Diseases. J Clin Immunol. 2017 Jul 1;37(5):486–95.

12. Piątosa B, Wolska-Kuśnierz B, Pac M, Siewiera K, Gałkowska E, Bernatowska E. B cell subsets in healthy children: reference values for evaluation of B cell maturation process in peripheral blood. Cytometry B Clin Cytom. 2010 Nov;78(6):372–81.

13. Duchamp M, Sterlin D, Diabate A, Uring-Lambert B, Guérin-El Khourouj V, Le Mauff B, et al. B-cell subpopulations in children: National reference values. Immunity, Inflammation and Disease. 2014;2(3):131–40.

14. Schatorjé EJH, Gemen EFA, Driessen GJA, Leuvenink J, van Hout RWNM, van der Burg M, et al. Age-matched reference values for B-lymphocyte subpopulations and CVID classifications in children. Scand J Immunol. 2011 Nov;74(5):502–10.

15. Huck K, Feyen O, Ghosh S, Beltz K, Bellert S, Niehues T. Memory B-cells in healthy and antibody-deficient children. Clin Immunol. 2009 Apr;131(1):50–9.

16. Morbach H, Eichhorn EM, Liese JG, Girschick HJ. Reference values for B cell subpopulations from infancy to adulthood. Clin Exp Immunol. 2010 Nov;162(2):271–9.

Figure S1 Flowchart of the patient selection for inborn error of immunity (IEI) cohort

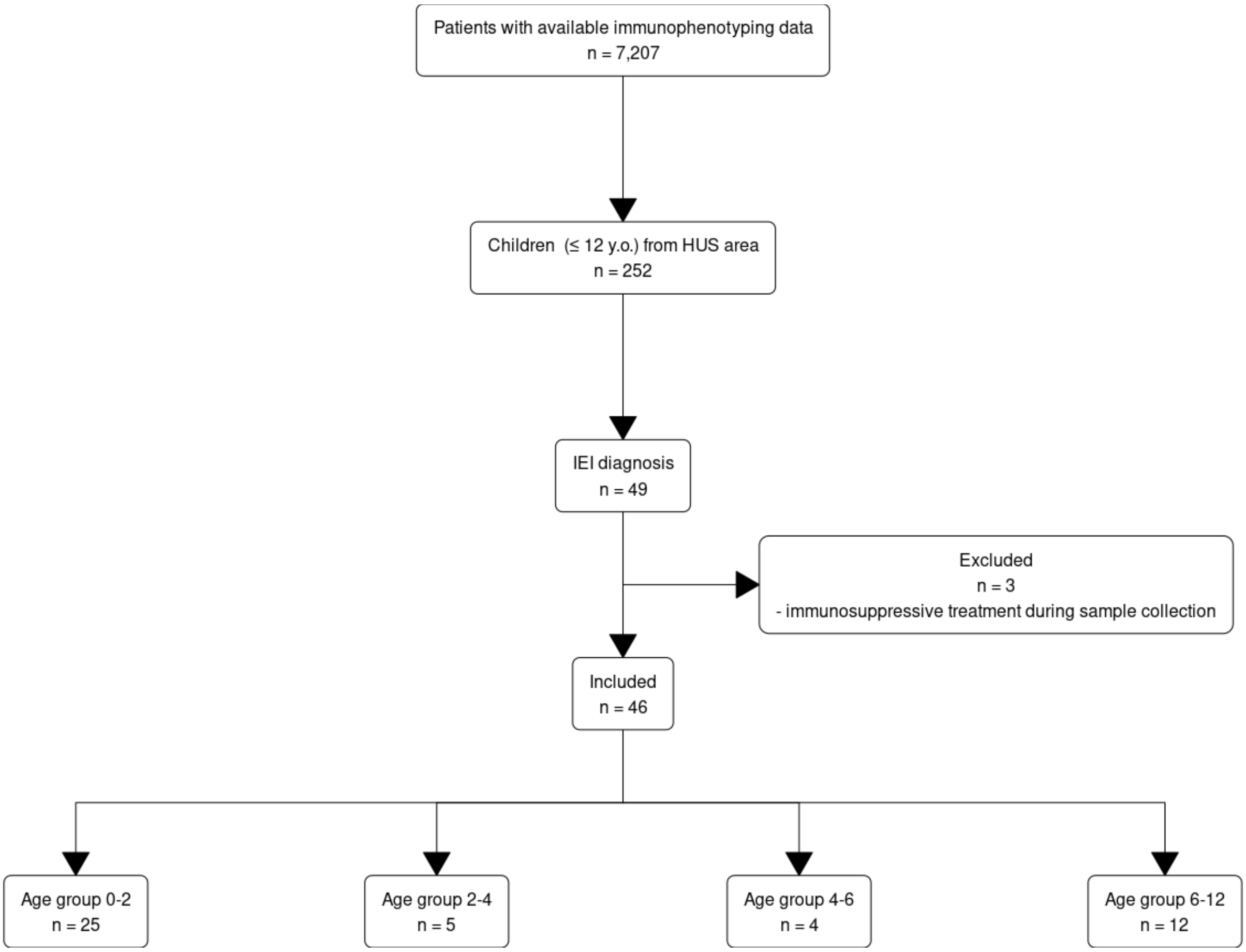

Table S1 Lymphocyte and dendritic cell definitions by cell surface markers used in HUS Diagnostic Center, Clinical Microbiology & Flow Cytometry, Helsinki, Finland

Main cell population	Subpopulation	Definition by cell surface markers (CD)
**T lymphocytes**	Total T	45/3
	Helper T	45/3/4
	Cytotoxic T	45/3/8
	Naïve (total, helper, cytotoxic)	45/3/45RA/CCR7<br>45/3/4/45RA/CCR7<br>45/3/8/45RA/CCR7
	Recent thymic emigrant (helper-RTE)	45/3/4/45RA/62L/31
	$CD45RA^{+}CD62L^{+}$ T cell	45/3/4/45RA/62L
	Regulatory T (Treg)	45/3/4/$CD25_{high}$/$CD127_{low}$
	Memory T	45/3/45RO
	Central memory T, TCM (helper, cytotoxic)	45/3/4/45RA−/CCR7<br>45/3/8/45RA−/CCR7
	Effector memory T, TEM (helper, cytotoxic)	45/3/4/45RA−/CCR7−<br>45/3/8/45RA−/CCR7−
	Terminally differentiated effector memory T, TEMRA (helper, cytotoxic)	45/3/4/45RA/CCR7−<br>45/3/8/45RA/CCR7−
	Activated T (total, helper, cytotoxic)	45/3/HLA-DR/38<br>45/3/4/HLA-DR/38<br>45/3/8/HLA-DR/38
	TCRαβ	45/TCRγδ−/3
	TCRγδ	45/TCRγδ/3
	Double-negative TCRαβ positive T, DNT	45/TCRαβ/3/4−/8−
	Double-positive T, DPT	45/3/4/8
**B lymphocytes**	Total B cell	45/19
	Naïve B	45/19/27−/IgD/IgM
	Memory B	45/19/27
	Switched memory B (smB)	45/19/IgD−/IgM−
	Non-switched memory B (nsmB)	45/19/IgD/IgM
	Activated B	45/19/21_{low}/38_{low}
	Transitional B	45/19/38/IgM
	Plasmablasts	45/19/27/38/IgM−
**Dendritic cells**	Plasmacytoid dendritic cell (pDC)	303/123/HLA-DR/11c−/56−/3−
	Myeloid dendritic cell (mDC)	11c/HLA-DR/123−/56−/3−/14−/19−

Table 2 Monoclonal antibodies, fluorochromes and clones used in HUS Diagnostic Center, Clinical Microbiology & Flow Cytometry, Helsinki, Finland

Panel	Monoclonal antibody	Fluorochrome	Clone	Brand
**T lymphocyte subpopulations**	CD3	PerCPCy5.5	SK7	Becton Dickinson (BD)
	CD4	APC-R700	SK3	BD
	CD8	APC-H7	SK1	BD
	CD25	PE	2A3	BD
	CD31	BV421	WM59	BD
	CD38	APC	HB7	BD
	CD45	Krome Orange	J33	Beckman Coulter
	CD45	V500-C	2D1	BD
	CD45RA	FITC	L48	BD
	CD45RO	PE-Cy7	UCHL1	BD
	CD56	BV605	NCAM16.2	BD
	CD62L	APC	DREG-56	BD
	CD197	PE	150503	BD
	Anti-TCR γ/δ	PE-Cy7	11F2	BD
	HLA-DR	FITC	L243	BD
**B lymphocyte subpopulations**	CD3	APC-Alexa Fluor 750	UCHT1	Beckman Coulter
	CD16	BV605	3G8	BD
	CD19	PE-Cy7	SJ25C1	BD
	CD21	PE	B-ly4	BD
	CD27	BV421	M-T271	BD
	CD38	PerCP-Cy5.5	HIT2	BD
	CD45	V500-C	2D1	BD
	CD56	BV605	NCAM16.2	BD
	IgD	FITC	IA6-2	BD
	IgM	APC	G20-127	BD
**Dendritic cells**	CD3	PerCP-Cy5.5	SK7	BD
	CD3	PerCP-Cy5.5	NCAM16.2	BD
	CD4	APC-H7	SK3	BD
	CD11c	APC	S-HCL-3	BD
	CD14	FITC	MϕP-9	BD
	CD19	FITC	SJ25C1	BD
	CD45	Krome Orange	J33	Beckman Coulter
	CD56	PE-Cy7	NCAM16.2	BD
	CD123	PE	9F5	BD
	CD303	FITC (BDCA-2)	AC144	MACS Miltenyi
	cyMPO-7	FITC	MPO-7	Dako
	HLA-DR	V450	L243	BD
**FASCIA**	CD3	FITC	SK7	BD
	CD4	PE	SK3	BD
	CD8	PerCP-Cy5.5	SK1	BD
	CD19	APC	SJ25C1	BD

Figure S2 Strategy used in gating T cell subsets. CD4+ and CD8+ memory and naïve cells were gated from their parent gates similarly than the CD3+ memory and naïve cells shown in the figure

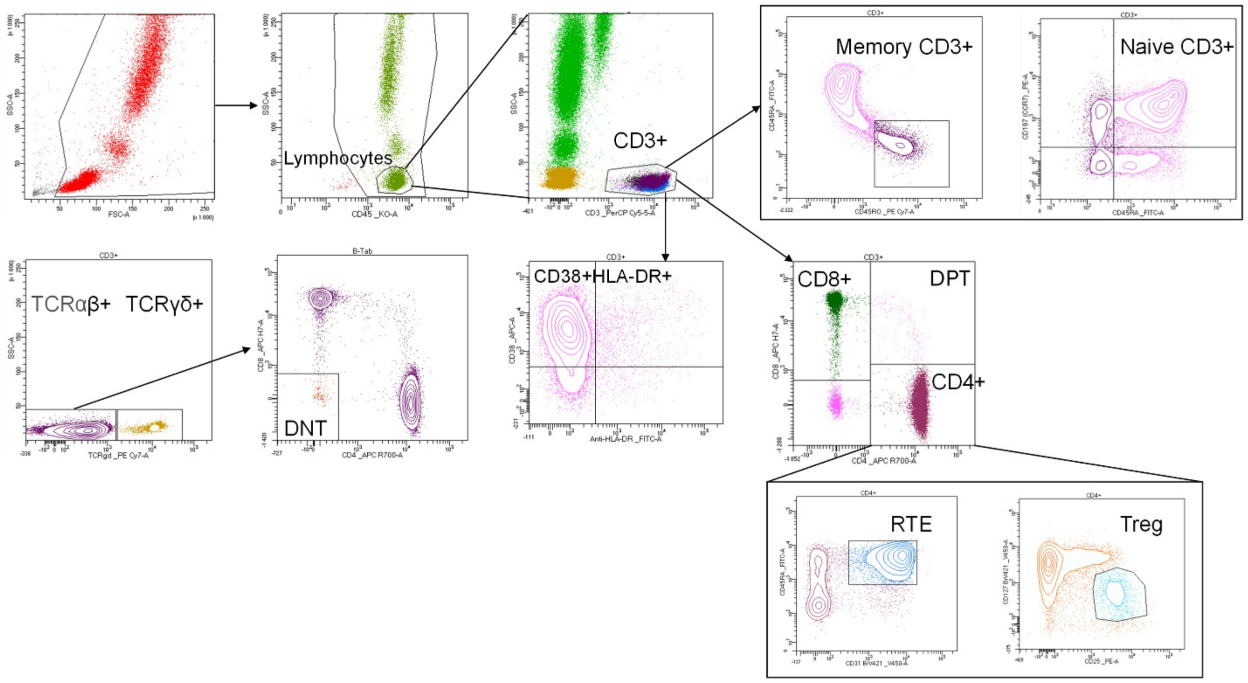

Figure S3 Strategy used in gating B cell subsets

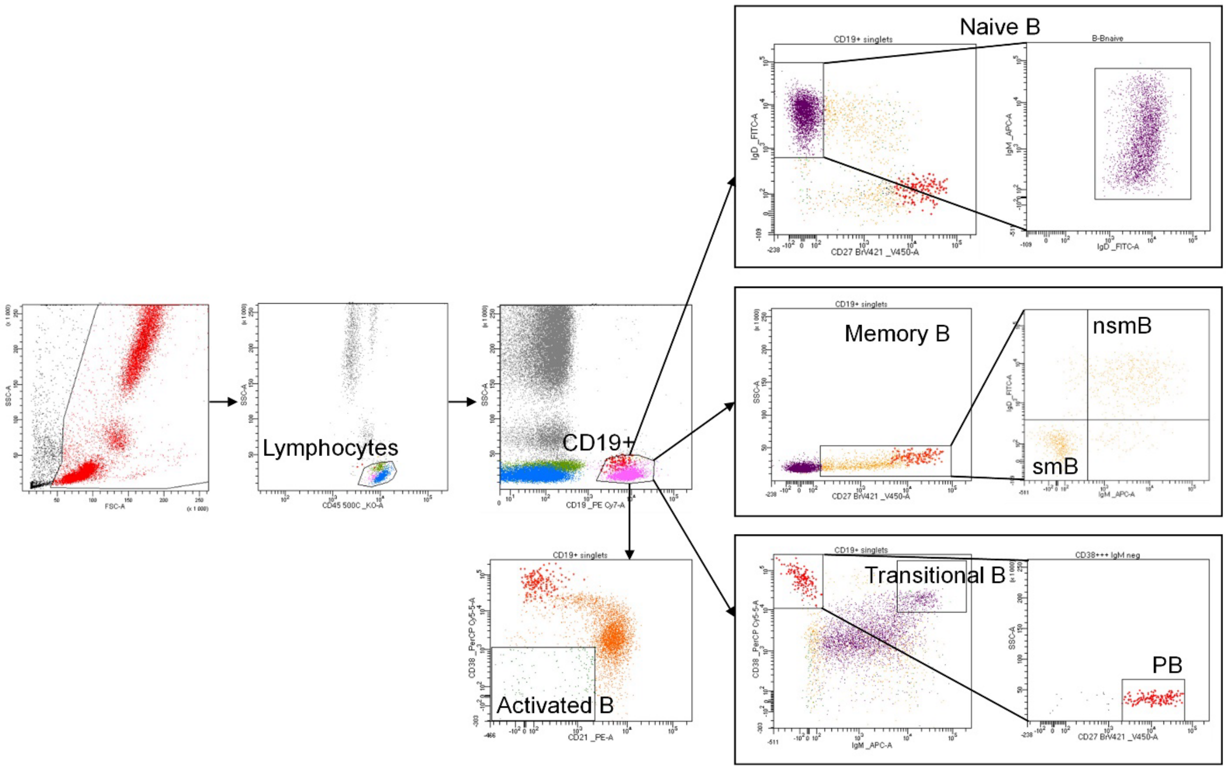

Figure S4 Strategy used in gating dendritic cell subsets

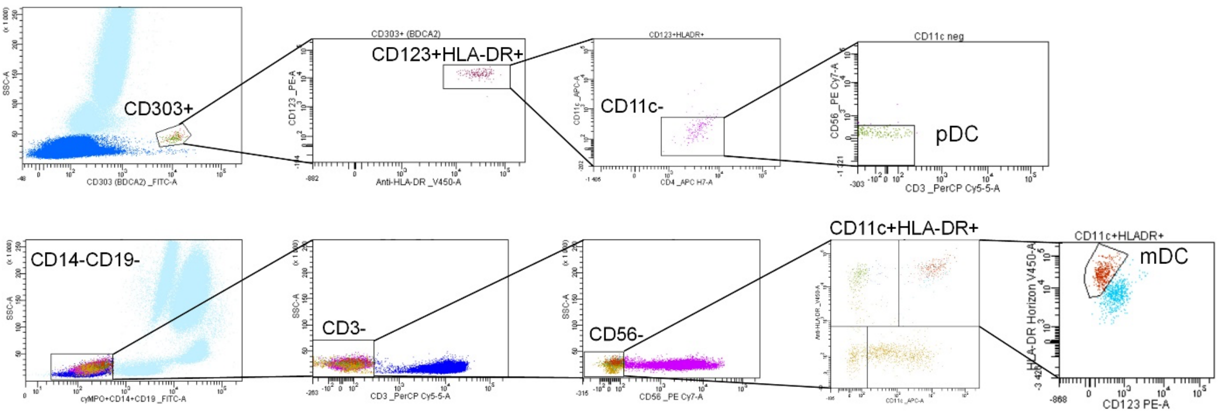

Figure S5 Comparison of absolute values provided by calculation from Sysmex (XN-2000) hematological analyzer and from flow cytometric BD FACSLyric system with Trucount tubes (BD Multitest reagents). Statistical analysis with paired Wilcoxon signed rank test showed statistically significant differences with absolute values provided by calculation from Sysmex analyzer and flow cytometric BD FACSLyric system with Trucount tubes. P-values were < 0.001 in all examined cell populations (total, CD3+, CD4+, and CD8+ T cells). Flow cytometric analysis provided higher values than calculation from Sysmex analyzer data

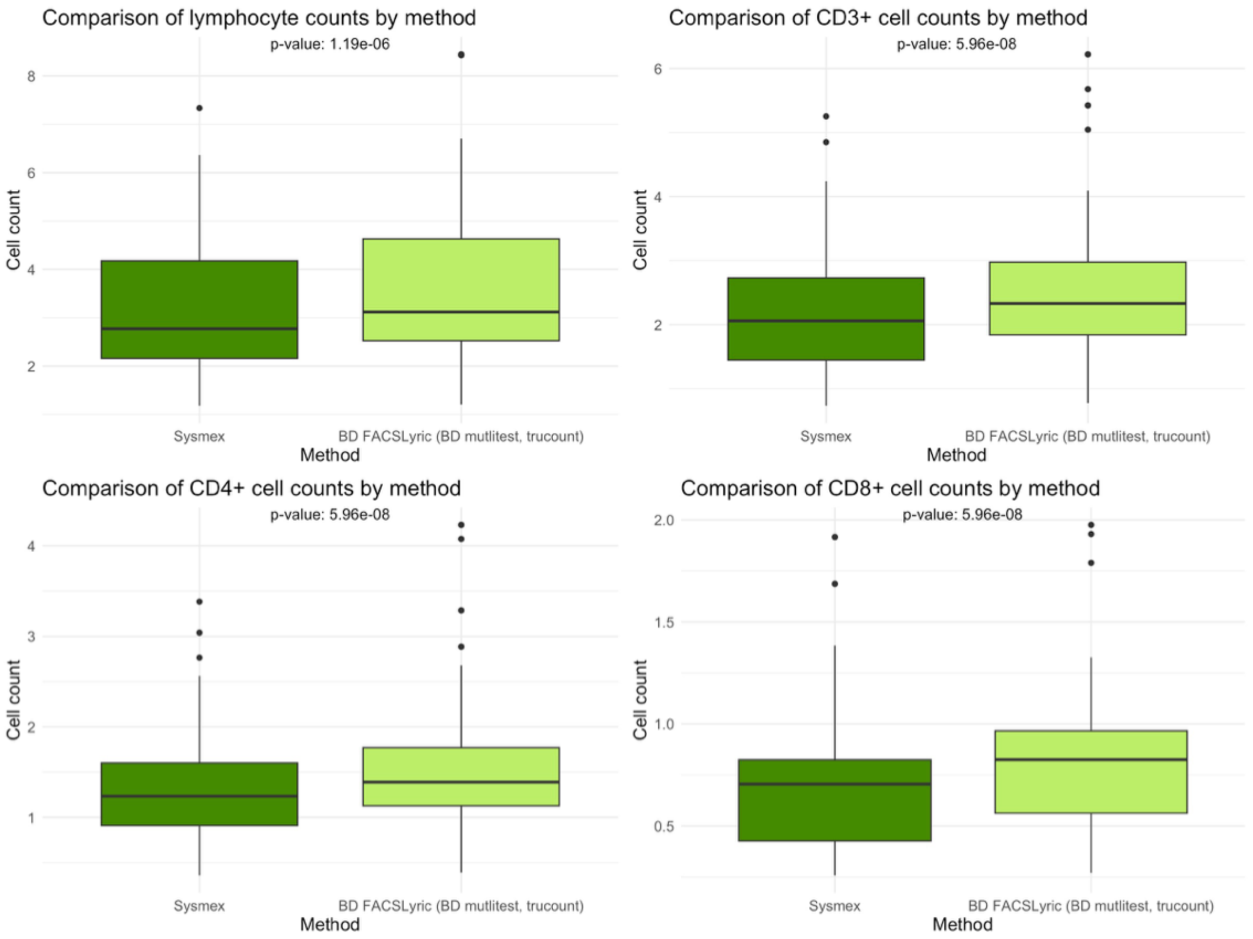

Table S3 Previously published reference values for pediatric extended immunophenotyping of T cells. The table showcases used statistical interval, age cohorts, and lymphocyte subpopulation definitions (cell-surface markers, CD)

Article	Statistical Interval	Age Cohorts	T cell subpopulation definitions				
			RTE	Naïve CD4+ or CD8+	CD8+ TEMRA	DNT	γδ T
van Gent 2009	Value Range	0–6 mth, 6–12 mth, 1–2 y, 2–3 y, 3–4 y, 4–6 y, 6–9 y, 9–12 y	4/45RO–/27/31	4 or 8 /45RO–/27	N/A	N/A	N/A
Tosato 2014	10–90 PR	0–3 mth, 3–12 mth, 1–2 y, 2–6 y, 6–12 y	N/A	N/A	N/A	N/A	3/TCRγδ
Shearer 2003	10–90 PR	0–3 mth, 3–6 mth, 6–12 mth, 1–2 y, 2–6 y, 6–12 y	N/A	3/ 4 or 8 /45RA/62L+/–	N/A	N/A	N/A
Schatorjé 2011	0.90 TI	1 wk – 2 mth, 2–5 mth, 5–9 mth, 9–15 mth, 15–24 mth, 2–5 y, 5–10 y	3/4/45RA/31	3/4/45RA/27 and 3/8/45RA/197(=CCR7)/27	3/8/45RA/197(=CCR7)–/27–	3/4–/8–/TCRαβ	3/TCRγδ
Moraes-Pinto 2014	10–90 PR	0–3 mth, 3–6 mth, 6–12 mth, 1–2 y, 2–6 y, 6–12 y	N/A	3/ 4 or 8 /45RA/27	3/8/45RA/CCR7–	N/A	N/A
Lerkvaleekul 2020	25–75 PR [IQR]	0–2 y, 2–5 y, 5–10 y	N/A	N/A	N/A	45/3/4–/8–[1]	45/3/4–/8–/TCRγδ
Kavgaci 2023	5–95 PR	6 mth, 1 y, 2 y, 4 y, 6 y	4/45RA/31	N/A	N/A	N/A	N/A
Jodhawat 2023	10–90 PR	0–6 mth, 6–12 mth, 1–2 y, 2–5 y, 5–10 y	45/3/4/45RA/62L/31	45/3/ 4 or 8 /45RA/27	45/3/8/45RA/62L–	3/4–/8–/TCRαβ	3/TCRγδ
Garcia-Prat 2018	10–90 PR	0–2 y, 3–4 y, 5–6 y, 7–8 y, 9–13 y	45/3/4/45RA/27/62L/31	45/3/45RA/CCR7	45/3/45RA/CCR7–	N/A	N/A
Besci 2021	0.90 TI	0–39 d, 40 d – 6 mth, 6–9 mth, 9–12 mth, 1–2 y, 2–5 y, 5–10 y	3/4/45RA/31	3/4/45RA/27 and 3/8/45RA/CCR7[2]	3/8/45RA/CCR7–	3/4−/8−/TCRαβ?[3]	3/TCRγδ
Takashima 2017	SD (and mean)	0–1 y, 2–6 y, 7–19 y	3/4/45RA/31[4]	3/ 4 or 8 /45RA/45RO–[2]	N/A	3/4–/8–/TCRαβ[5]	3/TCRγδ
Sagnia 2011	10–90 PR	0–3 mth, 3–6 mth, 6–12 mth, 12–24 mth, 2–6 y	N/A	3/ 4 or 8 /45RA/62L+/–	N/A	N/A	3/TCRγδ/TCRαβ–[6]
Ding 2018	10–90 PR	1–6 mth, 6–12 mth, 1–4 y, 4–8 y, 8–12 y	N/A	3/ 4 or 8 /45RA/27	3/8/45RA/27–	3/4–/8–/TCRαβ	3/TCRγδ

[1] *Double-negative cells include TCRγδ+ cells (thus not DNTs).*
[2] *Absolute values are not provided.*
[3] *Does not unequivocally specify, whether DNTs include TCRγδ+ cells.*
[4] *% of CD3+ CD4+ CD45RA+ T cells*
[5] *% of CD3+ TCRαβ+ T cells*
[6] *% of total lymphocytes*
RTE, recent thymic emigrant; TEMRA, terminally differentiated memory effector T cell; DNT, double-negative T cell (TCRαβ+); γδ T, gamma-delta T cell
PR, percentile range; TI, tolerance interval; IQR, interquartile range; SD, standard deviation
N/A, not available

Table S4 Previously published reference values for pediatric extended immunophenotyping of B cells. The table showcases used statistical interval, age cohorts, and lymphocyte subpopulation definitions (cell-surface markers, CD)

Article	Statistical Interval	Age Cohorts	B cell subpopulation definitions						
			Naïve B	Memory B	nsmB	smB	Activated B	Transitional B	Plasmablasts
van Gent 2009	Value Range	0–6 mth, 6–12 mth, 1–2 y, 2–3 y, 3–4 y, 4–6 y, 6–9 y, 9–12 y	19/27–/38–/IgD	N/A	19/27/IgD/ IgM	19/27/IgD– /IgM–[1]	N/A	N/A	N/A
Schatorjé 2011	TI 0.90	2–5 mth, 5–9 mth, 9–15 mth, 15–24 mth, 2–5 y, 5–10 y	19/27–/IgD/IgM	N/A	19/27/IgD/IgM	19/27/IgD– /IgM–	19/21^{low}/38^{low}	19/38^{high}/IgM^{high}	19/38^{high}/IgM–
Piatosa 2010	5–95 PR	2–5 mth, 5–9 mth, 9–15 mth, 15–24 mth, 2–5 y, 5–10 y	19/27–/IgD	19/27	19/27/IgD	19/27/IgD–	19/21^{low}/38^{low}	19/38^{high}/IgM^{high}	19/38^{high}/IgM–
Morbach 2010	25–75 PR [IQR]	0–1 y, 2–3 y, 4–5 y, 6–10 y	19/27–/IgD	N/A	19/27/IgD	19/27/IgD–	19/21^{low}/38^{low}	19/24^{high}/38^{high}	19/24–/38^{high}
Huck 2009	10–90 PR	1 y, 2–3 y, 4–5 y, 6–10 y	20/27–/IgD	N/A	20/27/IgD	20/27/IgD–	N/A	N/A	N/A
Garcia-Prat 2018	10–90 PR	0–2 y, 3–4 y, 5–6 y, 7–8 y, 9–13 y	19/27–/IgD	N/A	19/27/IgD	19/27/IgD–	19/21^{low}	19/27–/24/38/IgD	19/27/24–/38/IgD–
Duchamp 2014	5–95 PR	1–6 mth, 6–18 mth, 18 mth – 4 y, 4–8 y, 8–12 y	19/27–/IgD	19/27	19/27/IgD	19/27/IgD–	N/A	N/A	N/A
Besci 2021	TI 0.90	0–39 d, 40 d – 6 mth, 6–9 mth, 9–12 mth, 1–2 y, 2–5 y, 5–10 y	19/27–/IgD	N/A	19/27/IgD	19/27/IgD–	19/21^{low}/38^{low}	N/A	N/A
Berrón-Ruíz 2016	5–95 PR	1–24 mth, 3–5 y, 6–10 y	19/27–/IgD	19/27	19/27/IgD	19/27/IgD–	19/21^{low}/38^{low}[2]	19/24^{high}/38^{high}	19/24–/38^{high}
Jodhawat 2023	10–90 PR	0–6 mth, 6–12 mth, 1–2 y, 2–5 y, 5–10 y	19/27–/IgD	19/27	19/27/IgD/IgM	19/27/IgD– /IgM–	N/A	N/A	N/A
Takashima 2017	SD (and mean)	0–1 y, 2–6 y, 7–19 y	19/27–/24/38^{low}[3]	19/27/24/38−	19/27/IgD/IgM	19/27/IgD– /IgM–	N/A	19/24^{high}/38^{high}/IgM	19/24–/38^{high}/IgM–
Ding 2018	10–90 PR	1–6 mth, 6–12 mth, 1–4 y, 4–8 y, 8–12 y	19/27–/IgD	19/27/IgD–	N/A	N/A	N/A	19/24^{high}/38^{high}	19/24–/38^{high}

[1]*Values only provided for 19/27/IgD–/IgM population which were used in the analysis.*
[2]*Not assessed in the paediatric cohort.*
[3]*Values not provided.*
nsmB, non-switched memory B cell; smB, switched memory B cell
TI, tolerance interval; PR, percentile range; IQR, interquartile range
N/A, not available

Table S5 The number of patient samples for each T cell subset in IEI cohorts

**T cell subset**	**IEI cohort**	**n**
CD8+ TEMRA	22q11.2del	13
	CVID/Antibody deficiency	6
	CHH	6
	CID	11
	HIES	6
DNT	22q11.2del	13
	CVID/Antibody deficiency	6
	CHH	6
	CID	11
	HIES	6
Naive CD4+	22q11.2del	13
	CVID/Antibody deficiency	6
	CHH	6
	CID	11
	HIES	6
Naive CD8+	22q11.2del	13
	CVID/Antibody deficiency	6
	CHH	6
	CID	11
	HIES	6
RTE	22q11.2del	13
	CVID/Antibody deficiency	6
	CHH	6
	CID	11
	HIES	6
γδ T	22q11.2del	13
	CVID/Antibody deficiency	6
	CHH	6
	CID	11
	HIES	6

IEI, inborn error of immunity; TEMRA, terminally differentiated effector memory cell; DNT, TCRαβ+ double-negative T cell; RTE, recent thymic emigrant; 22q11.2del, 22q11.2 deletion syndrome; CVID, common variable immunodeficiency; CHH, cartilage-hair hypoplasia; CID, combined immunodeficiency; HIES, hyperimmunoglobulin E syndrome

Table S6 The number of patient samples for each B cell subset in IEI cohorts

**B cell subset**	**IEI cohort**	**n**
Activated B	CVID/Antibody deficiency	5
	CID	8
	HIES	< 5
Naive B	CVID/Antibody deficiency	5
	CID	8
	HIES	< 5
Non-switched memory B	CVID/Antibody deficiency	5
	CID	7
	HIES	< 5
Plasmablasts	CVID/Antibody deficiency	5
	CID	8
	HIES	< 5
Switched memory B	CVID/Antibody deficiency	5
	CID	8
	HIES	< 5
Total memory B	CVID/Antibody deficiency	5
	CID	8
	HIES	< 5
Transitional B	CVID/Antibody deficiency	5
	CID	8
	HIES	< 5

IEI, inborn error of immunity; CVID, common variable immunodeficiency; CID, combined immunodeficiency; HIES, hyperimmunoglobulin E syndrome
